# Hypoxia-treated adipose mesenchymal stem cell-derived exosomes attenuate lumbar facet joint osteoarthritis

**DOI:** 10.1186/s10020-023-00709-3

**Published:** 2023-09-05

**Authors:** Jinyun Zhao, Yi Sun, Xiaolong Sheng, Jiaqi Xu, Guoyu Dai, Rundong He, Yuxin Jin, Zhide Liu, Yong Xie, Tianding Wu, Yong Cao, Jianzhong Hu, Chunyue Duan

**Affiliations:** 1 Department of Spine Surgery and Orthopaedics, Xiangya Hospital, Central South University, Changsha, Hunan 410008 China; 2 Key Laboratory of Organ Injury, Aging and Regenerative Medicine of Hunan Province, Changsha, 410008 China; 3 Hunan Engineering Research Center of Sports and Health, Changsha, China; 4 National Clinical Research Center for Geriatric Disorders, Xiangya Hospital, Central South University, Changsha, 410008 China

**Keywords:** Adipose mesenchymal stem cells, Hypoxia-treated ADSC-derived exosomes, Lumbar facet joint osteoarthritis, Subchondral bone

## Abstract

**Background:**

Lumbar facet joint osteoarthritis (LFJ OA) is a common disease, and there is still a lack of effective disease-modifying therapies. Our aim was to determine the therapeutic effect of hypoxia-treated adipose mesenchymal stem cell (ADSC)-derived exosomes (Hypo-ADSC-Exos) on the protective effect against LFJ OA.

**Methods:**

The protective effect of Hypo-ADSC-Exos against LFJ OA was examined in lumbar spinal instability (LSI)-induced LFJ OA models. Spinal pain behavioural assessments and CGRP (Calcitonin Gene-Related Peptide positive) immunofluorescence were evaluated. Cartilage degradation and subchondral bone remodelling were assessed by histological methods, immunohistochemistry, synchrotron radiation-Fourier transform infrared spectroscopy (SR-FTIR), and 3D X-ray microscope scanning.

**Results:**

Hypoxia enhanced the protective effect of ADSC-Exos on LFJ OA. Specifically, tail vein injection of Hypo-ADSC-Exos protected articular cartilage from degradation, as demonstrated by lower FJ OA scores of articular cartilage and less proteoglycan loss in lumbar facet joint (LFJ) cartilage than in the ADSC-Exo group, and these parameters were significantly improved compared to those in the PBS group. In addition, the levels and distribution of collagen and proteoglycan in LFJ cartilage were increased in the Hypo-ADSC-Exo group compared to the ADSC-Exo or PBS group by SR-FTIR. Furthermore, Hypo-ADSC-Exos normalized uncoupled bone remodelling and aberrant H-type vessel formation in subchondral bone and effectively reduced symptomatic spinal pain caused by LFJ OA in mice compared with those in the ADSC-Exo or PBS group.

**Conclusions:**

Our results show that hypoxia is an effective method to improve the therapeutic effect of ADSC-Exos on ameliorating spinal pain and LFJ OA progression.

**Supplementary Information:**

The online version contains supplementary material available at 10.1186/s10020-023-00709-3.

## Introduction

Low back pain (LBP) is a common musculoskeletal disease that places heavy economic burdens on families and society (Maher and Ferreira 2021). In the United States, the economic cost associated with LBP exceeds $100 billion annually (Blyth et al. 2019). The lifetime prevalence of LBP is also very high and nearly 80% worldwide (Hayden et al. 2021). Unfortunately, the pathogenesis of LBP is still not fully understood, and there is a lack of effective disease-modifying treatments for this condition. In addition to intervertebral disc degeneration, lumbar facet joint osteoarthritis (LFJ OA) is a clinically important cause of LBP (O’Leary et al. 2018). The facet joints, which are the only synovial joints in the spine, are formed by the superior articular processes and inferior articular processes of adjacent vertebrae (Nakamura et al. 2019). Facet joints are anatomically and functionally distinct from the fibrocartilaginous articulation of the intervertebral disc (Rahimzadeh et al. 2017). The prevalence of LFJ OA increases with age, and the estimation of LFJ OA in the elderly population with LBP is approximately 40–85% (Goode et al. 2021). The clinical manifestations of LFJ OA generally include progressively localized back pain with a certain degree of radiation into the lower limbs (Gellhorn et al. 2013). Since pain is generated by nociceptors, LBP due to LFJ OA may be caused by sensory innervation within and surrounding LFJs (Groen et al. 1990). Prolonged peripheral inflammation in LFJs and their capsules can result in central sensitization and the progression of chronic spinal pain (Cavanaugh et al. 1997; Cohen et al. 2013). Compared with other OA phenotypes in large joints such as the knee, LFJ OA has received far less critical study and is often ignored in discussions of the effects on disability and function. Clarifying the pathological changes in LFJ OA is critical for determining prevention and treatment strategies.

Current research suggests that LFJ OA, which is a failure of the whole joint, is not as simple as the degeneration of facet joint cartilage (Kalichman and Hunter 2007). Emerging evidence has demonstrated that the pathologic changes in osteoarthritis (OA) joints include the degradation of all joint tissues, including the synovium, articular cartilage, and subchondral bone (Gellhorn et al. 2013). Articular cartilage homeostasis and integrity can lead to changes in joint load distribution and affect bone remodelling of the underlying subchondral bone (Cui et al. 2016), which stimulates nociceptor fibre innervation within subchondral bone followed by central sensitization and chronic spinal pain progression (Gellhorn et al. 2013). In turn, uncoupled bone remodelling in subchondral bone can promote cartilage degeneration (Zhang and Wen 2021). Furthermore, it is widely accepted that bone–cartilage could act as a functional unit and adjust their architecture to adaptations in response to the altered mechanical environment (Hu et al. 2021). In addition, abnormal vascular regrowth in subchondral bone was observed in OA joints (Wu et al. 2020). Recent studies have reported a novel H-type blood vessel that is increased in subchondral bone in OA, which can couple angiogenesis with osteogenesis (Peng et al. 2020). However, the role of subchondral bone remodelling and H-type vessel formation in LFJ OA remains to be further elucidated.

In recent years, exosome-based regenerative therapies have gained significant attention in basic and clinical research (Akbari et al. 2020). An increasing number of studies have demonstrated that exosomes derived from stem cells have the same effect as stem cell transplantation in protecting against OA progression (Chang et al. 2018). Exosomes are nanoscale membrane-bound vehicles with diameters of 50–150 nm that are resistant to degradation, have excellent characteristics, and provide a promising option for the treatment of OA (Meldolesi 2018). Unlike bone marrow, adipose tissue may represent an optimal source of stem cells that are easy to collect and is a much less painful procedure than harvesting bone marrow stem cells (Bacakova et al. 2018). In addition, adipose tissue is far richer in stem cells than bone marrow aspirate (Shin et al. 2020). Therefore, the current study used white adipose tissue-derived stem cells (ADSCs) from the mouse groin to isolate exosomes and explore the effect of ADSC-Exos on LFJ OA.

The articular cavity is in a negative pressure microenvironment of micro hypoxia. Inflammatory reactions or tissue damage increase oxygen consumption, resulting in a hypoxic microenvironment in the joint articular cavity (Lu et al. 2021). It has been reported that the oxygen level in cell culture has a significant effect on the proliferation, differentiation, and self-renewal of MSCs (Yang et al. 2021). Under in vitro culture conditions, MSCs are typically exposed to normoxia (21% O_2_), which is very different from the hypoxic environment associated with osteoarthritis in vivo (Hu and Li 2018). It has been reported that hypoxic ADSC-Exos have higher levels of growth factors than normoxic ADSC exosomes (Han et al. 2019). However, it is unclear whether the protective effects of hypoxic ADSC-Exos against LFJ OA are different from those of normoxic ADSC-Exos.

Therefore, in this study, we examined the therapeutic effect of hypoxia-modified ADSC-Exos on an LFJ OA model and examined their protective effects on articular cartilage and subchondral bone and the amelioration of spinal pain caused by LFJ OA.

## Materials and methods

### Animals

C57BL/6 male mice (25–30 g, 8 weeks) were purchased from Vital River Laboratory Animal Technology Co. Ltd. (Beijing, China). The animal studies were approved by the Ethics Committee of A University (No: 2020sydw0374). All animal experimental protocols were in accordance with the National Institutes of Health Guide for the Care and Use of Laboratory Animals. The mice were housed in a specific pathogen-free facility and were provided ad libitum access to food and water throughout the study. Environmental conditions, including constant temperature (21–23 °C) and humidity (45–50%), were maintained.

### ADSC isolation and hypoxia protocol

Mouse ADSCs were isolated from white adipose tissue of the groin and cultured as previously described (Shang et al. 2015). For hypoxic culture, the oxygen concentration was kept at 1% with a residual gas mixture composed of 5% CO_2_ and balanced nitrogen.

### Exosome isolation and identification

Passage 3 ADSCs were cultured with complete DMEM containing exosome-free FBS under hypoxic or normoxic conditions (Hu et al. 2023). The normoxic and hypoxic exosomes were isolated from the culture medium with an exosome extraction kit (Exoquick-TC, System Biosciences, Mountain View, CA) according to the manufacturer’s protocol. The size and concentration of exosomes were determined by nanoparticle tracking analysis (NTA, ZetaView, Germany). Exosome morphology was measured by using transmission electron microscopy (TEM, HITACHI H-7000FA, Japan). Exosome surface marker proteins, including CD63, CD81, and TSG101, were examined by Western blotting.

### LFJ OA model induction

**Fig. 1:**
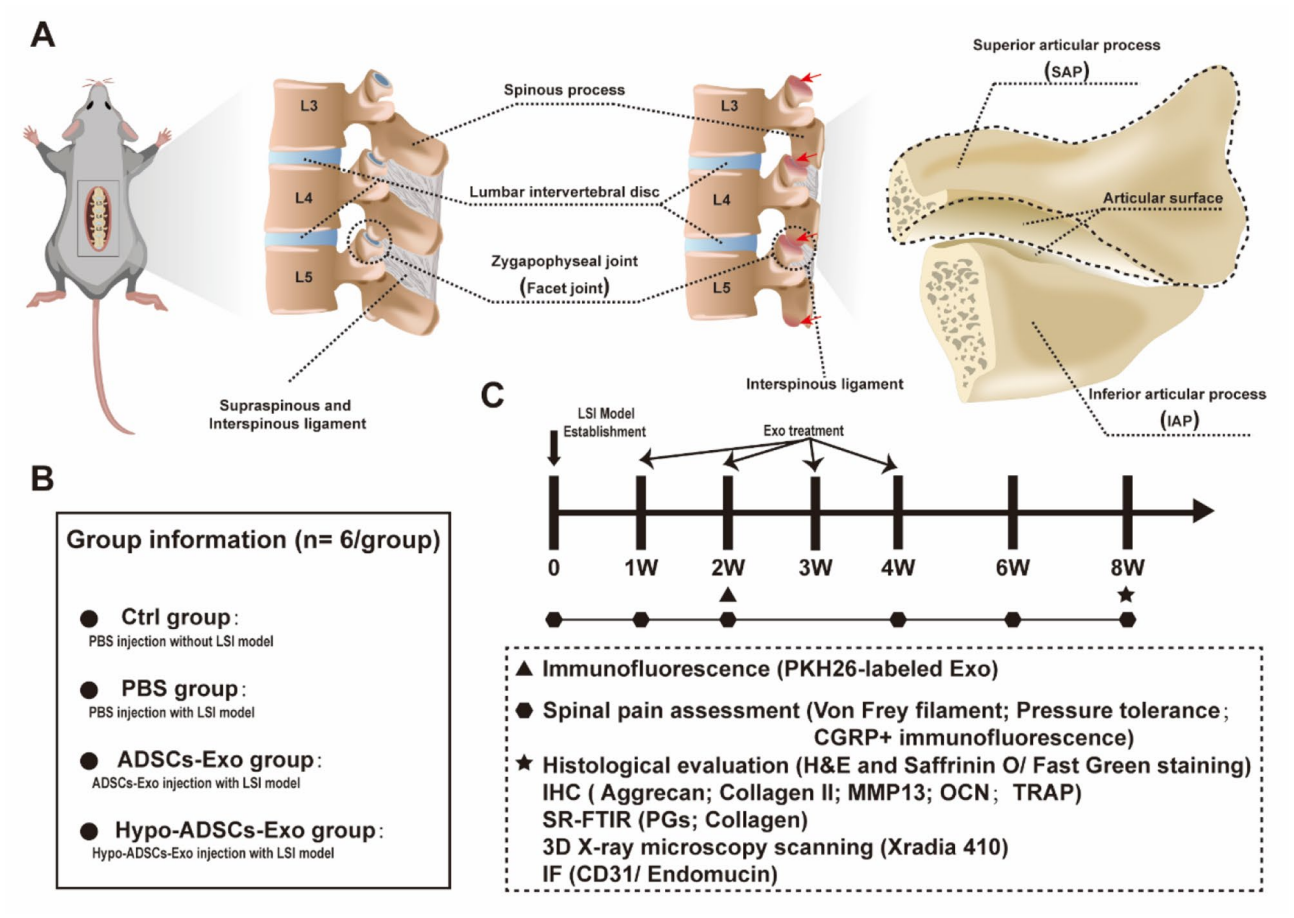
Schematic of lumbar spine instability (LSI) model establishment and diagram of timeline for examination postoperative among different treatment groups in vivo. (**A**) Schematic of LSI model establishment and red arrows indicate the facet joint osteoarthritis induced by LSI; (**B**) Group information and details; (**C**) Schedule time points for spinal pain tests, histological evaluation, immunohistochemistry, SR-FTIR, 3D X-ray microscopy analysis and immunofluorescence

The construction of the LFJ OA model was based on a previously reported method (Miyamoto et al. 1991; Bian et al. 2016; Ariga et al. 2001; Xue et al. 2021; Liu et al. 2021; Li et al. 2020a, b). A total of 102 8-week-old male mice were used to perform the experiments. Six mice per cage were kept for 1–8 weeks during the experimental period. Briefly, the mice were anesthetized by an intraperitoneal injection of 0.3% pentobarbital sodium (60 mg/kg), and the lumbar spine instability (LSI) mouse model was induced by resecting the lumbar 3rd − 5th (L3–L5) spinous processes along with supraspinous and interspinous ligaments without removing any elements in the vertebral canal (Fig. 1). Antibiotic administration (penicillin) was performed daily for 3 days after LSI surgery. Mice in the control group only underwent separation of the posterior paravertebral muscle from the lumbar 3rd − 5th vertebrae. As shown in Fig. 1, the mice were randomly divided into four groups: control group (PBS injection without LSI surgery), PBS group (PBS injection with LSI surgery), ADSC-Exo group (ADSC-Exo injection with LSI surgery), and Hypo-ADSC-Exo group (Hypo-ADSC-Exo injection with LSI surgery).

### Preparation and tracking of Hypo-ADSC-Exos and ADSC-Exos in vivo

The red PKH membrane dye PKH26 was used to label Hypo-ADSC-Exos, and then PKH26-labelled Hypo-ADSC-Exos (200 µg per mouse, once per week for 4 weeks) (Li et al. 2020a, b) were injected through the tail vein after surgery. To assess the relative distribution of exosomes within the cartilage or subchondral bone of L4/5 FJ, the sections were stained with 4′,6-diamidino-2-phenylindole (DAPI) 14 days after LSI and then examined with fluorescence imaging under a 40x objective lens.

### Spinal pain behavioural assessment

Several studies have shown that mechanical allodynia of the hind paw is a secondary indicator of spinal pain-related behaviours, which indicates that plantar pain is related to spinal pain (Shuang et al. 2015; Kim et al. 2011, 2015; Millecamps et al. 2015). Spinal pain behavioural evaluations were performed before and 1, 2, 4, 6, and 8 weeks after LFJ OA induction. Von Frey filaments (Stoelting, Wood Dale, IL) of two force levels (0.7 mN and 3.9 mN) were used to measure the hind paw withdrawal frequency in response to a mechanical stimulus. The mice were placed on a wire metal mesh grid, and von Frey filaments were applied to the midplantar surface of the hind paw. If paw withdrawal occurred, it was recorded. The mechanical withdrawal frequency was calculated as the percentage of withdrawal times in response to ten applications.

Vocalization thresholds in response to the force of an applied force gauge (SMALGO algometer; Bioseb, Pinellas Park, FL) were used to reflect spinal pain (Kim et al. 2011). A 5-mm-diameter sensor tip was pressed to the dorsal skin of the lumbar spine. The pressure force was increased 50 g/s until an audible vocalization was made. Two tests were performed, and the mean value was calculated as the pressure tolerance.

### Histological evaluation

At the time of euthanasia, the L4–L5 lumbar spines were fixed in 10% buffered formalin. Then, the spine samples were decalcified in 10% ethylenediaminetetraacetic acid (EDTA) (pH 7.4) for 14 days and embedded in paraffin. Four micrometre-thick sections of the L4–L5 lumbar spine were stained with haematoxylin-eosin (H&E) and Safranin O/Fast Green and examined by a light microscope under a 40x objective lens. A semiquantitative FJ OA scoring system was used to evaluate articular cartilage changes after different interventions as previously described (Wang et al. 2022). The synovitis assay was scored on an arbitrary scale from 0 to 3 depending on inflammatory cell infiltration into the synovium (0 = no inflammation; 1 = mild inflammation; 2 = moderate inflammation; and 3 = severe inflammation) (Midwood et al. 2009; Kang et al. 2019).

### Synchrotron radiation Fourier transform infrared (SR-FTIR) spectroscopy

Before SR-FTIR scanning, all FJ samples were prepared for fixation, decalcification, and cryosectioning as previously described. The 14 μm cryosections were placed onto the BaF2 substrate (Spectral Systems, Hopewell Junction, USA) and then examined by a Nicolet Continuum XL microscope (Thermo Fisher Scientific, Wilmington, MA, USA) coupled with a Nicolet 6700 spectrometer (Thermo Fisher). After a total of 32 scans, the spectra were acquired with a spectral resolution of 8 cm − 1 and an aperture of 20 × 20 μm. The maps were collected with OMNIC 9 software (Thermo Fisher Scientific). Raw spectra were baseline corrected and then smoothed by Savitzky‒Golay, and five points of each sample were selected randomly for semiquantitative analysis (Guo et al. 2020).

### 3D X-ray microscope scanning and image analysis

After the surgery, the lumbar segment (L4-L5) was harvested and fixed with 10% formalin at 4 °C and analysed by a 3D X-ray microscope at a high resolution of 4.75 μm/pixel. 3D X-ray microscope scans were acquired at the corresponding voltage (50 kV) and power (10 w). To quantitatively analyse the subchondral bone, the 3D structural parameters were systematically analysed.

### Immunofluorescence and immunohistochemistry

For immunofluorescence staining, decalcified lumbar segment (L4-L5) samples were embedded in optimal cutting temperature (OCT) compound (Sakura Finetek, Torrance, CA). Eight-micron-thick slices were treated with a Triton X-100 PBS solution (0.3%, w/v) and blocked with a goat serum PBS solution (10%, v/v) at room temperature. Then, the slices were incubated with CGRP (1:200, Abcam), CD31 (1:100, Abcam) and endomucin (1:50, Santa Cruz) primary antibodies overnight at 4 °C. Then, the corresponding secondary antibodies were added to the sections. DAPI was used to stain the nucleus, and images were captured by a fluorescence microscope (Olympus BX51, DP71). The decalcified spine samples were embedded in paraffin, sectioned into 4-µm-thick sections and incubated with primary antibodies against OCN (Abcam, 1:600, ab22552), Aggrecan (Abcam, 1:50, ab36861), MMP13 (Abcam, 1:50, ab3208), and collagen II (Abcam, 1:100, ab58632) followed by counterstaining with haematoxylin (Sigma‒Aldrich). Then, images were observed under a light microscope under a 40x objective lens. ImageJ (NIH) software was used for quantitative analysis. The number of positively stained cells was counted in the cartilage and subchondral bone areas of each LFJ specimen, and eight sequential specimens per mouse in each group were analysed (Li et al. 2020a, b).

### Statistical analysis

All statistical analyses were performed with SPSS software (version 25.0; SPSS Inc). The data are expressed as the mean ± standard deviation. For spinal pain behaviour testing between two groups, a two-way analysis of variance (ANOVA) with Tukey’s post hoc test was used. For comparisons between multiple groups, one-way analysis of variance (ANOVA) with Tukey’s post hoc test was used. For comparisons between two groups, unpaired two-tailed Student’s t tests were used. A value of *p* < 0.05 was defined as statistically significant. ‘^*^’ denotes the ADSC-Exo group compared with the PBS treated group, ‘^#^’ denotes the Hypo-ADSC-Exo group compared with the ADSC-Exo group.

## Results

### Identification of Hypo-ADSC-Exos and ADSC-Exos

**Fig. 2:**
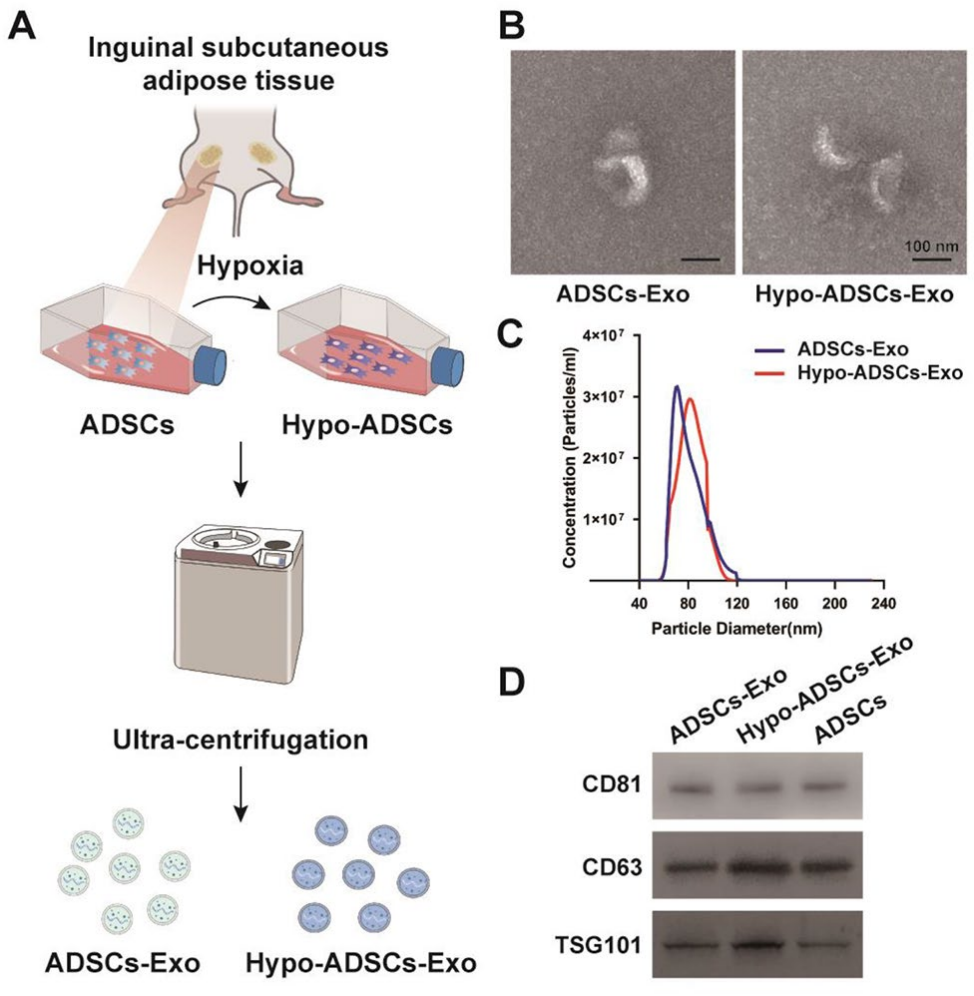
Identification of normoxia and hypoxia treated ADSCs-Exo. (**A**) The flow chart of isolation protocols for Hyo-ADSCs-Exo and ADSCs-Exo; (**B**) Morphology of ADSCs-Exo derived from hypoxic and normoxic conditions, as assessed by TEM; (**C**) The size distribution of Hypo-ADSCs-Exo and ADSCs-Exo; (**D**) Western blotting demonstrated the presence of exosomal surface markers CD81, CD63, and TSG101 between ADSCs-Exo and Hypo-ADSCs-Exo

The flow chart showing Hyo-ADSC-Exo isolation is depicted in Fig. 2A. The characteristics of ADSC-Exos under normoxic and hypoxic conditions were identified. Hypo-ADSC-Exos and ADSC-Exos exhibited consistent phenotypes. The TEM image showed that the isolated Hypo-ADSC-Exos exhibited a spherical morphology similar to normoxic ADSC-Exos (Fig. 2B). The diameters of Hypo-ADSC-Exos and ADSC-Exos showed similar size distributions, as measured by NTA (average 121.6 nm vs. 125.3 nm) (Fig. 2C). Exosomes expressed the same markers in the normoxia and hypoxia groups (Fig. 2D), including CD63, CD81, and TSG101, as identified by Western blotting.

### Hypo-ADSC-Exos alleviate symptomatic spinal pain-related behaviours

**Fig. 3:**
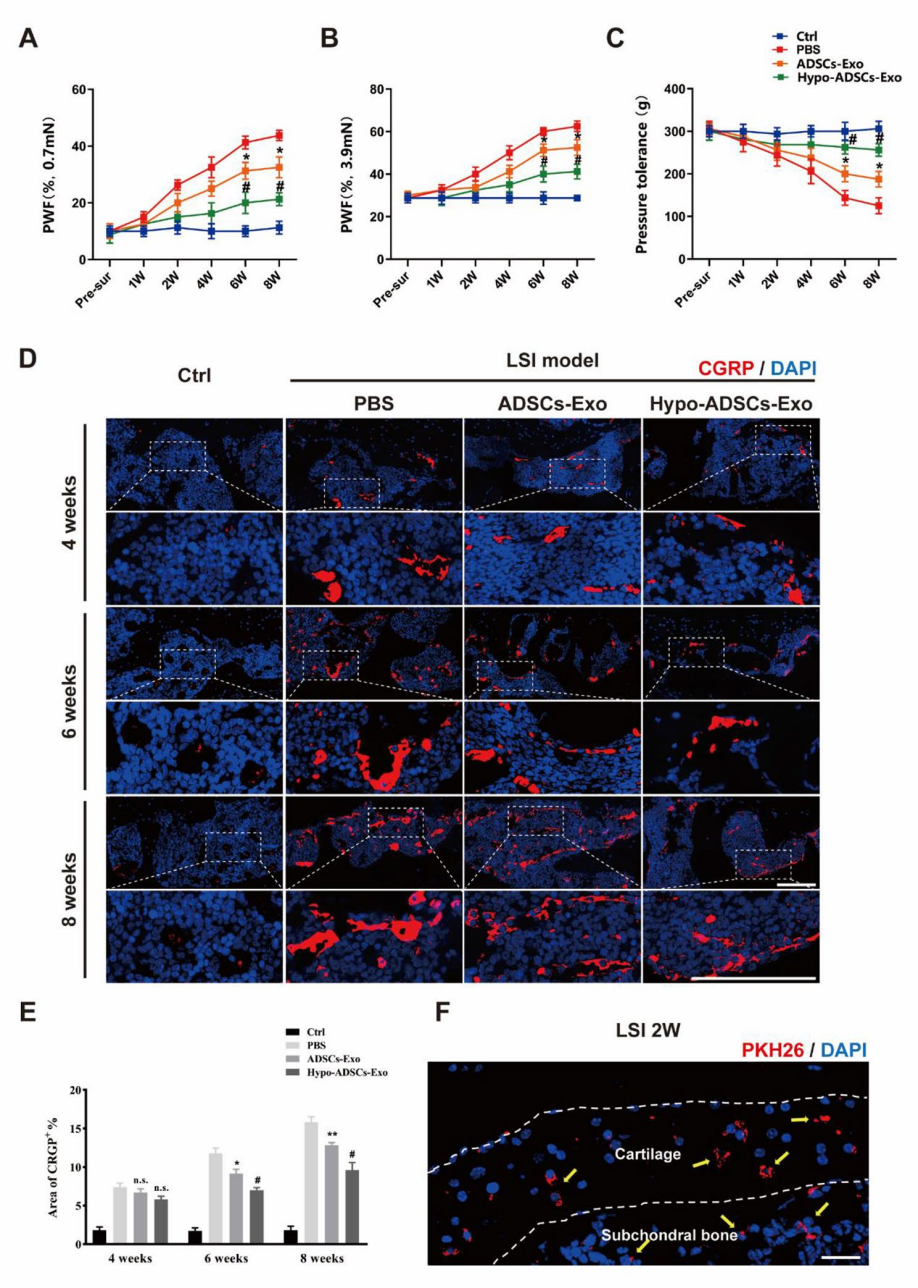
Immunofluorescence of CGRP^+^ and exosomes uptake and quantitative analysis of spinal pain-related behavior tests among different treatment groups. (**A–B**) The hind-paw withdrawal frequency (PWF) responding to the Von-Frey filaments with 0.7 mN and 3,9 mN; (**C**) Pressure hyperalgesia of the lumbar spine. (**D**) Representative images of immunofluorescence of CGRP+ (A marker of nociceptor nerves, Red-Alexa Fluor® 594) in subchondral bone of LFJ in vivo under 40x objective lens in 4, 6 and 8 weeks. Scale bar, 100 μm. (**E**) Quantitative analysis of the percentage of CGRP+ area in subchondral bone of LFJ. (**F**) Representative image of immunofluorescence of PKH26 labeled Hypo-ADSCs-Exo (PKH26^+^, Red) through tail vein administration in facet joint cartilage and subchondral bone of LFJ in vivo. The yellow arrows indicate that Hypo-ADSCs-Exo by administration of tail vein were internalized in cartilage zone and subchondral bone area. Scale bar, 100 μm. All images were captured under 40x objective lens. All data are shown as the mean ±standard deviation (SD). *p ＜ 0.05, **p ＜ 0.01, compared with PBS treated group mice, ^#^p ＜ 0.05, ^##^p ＜ 0.01, compared with ADSCs-Exo mice. n.s., non-significant. n=6 per group

Next, we evaluated the effect of Hypo-ADSC-Exos on LFJ OA in vivo (Fig. 1). First, we investigated whether Hypo-ADSC-Exos could effectively reduce pain in the LFJ OA model, and we examined their analgesic effect on LFJ OA by measuring the pressure tolerance and retraction frequency of the hind paw. Hypo-ADSC-Exos and ADSC-Exos were administered immediately after LSI model induction by tail vein injection. As shown in Fig. 3A-C, LSI surgery resulted in a significant increase in the retraction frequency of the hind paw and a decrease in pressure tolerance relative to those of sham mice. The pain behaviour tests indicated that LSI induced low back pressure hyperalgesia. In the first-round test 1, 2 and 4 weeks after exosome treatment, there was no significant difference in analgesic alleviation between the Hypo-ADSC-Exo-, ADSC-Exo- and PBS-treated groups. However, a significant analgesic effect was achieved in the ADSC-Exo group from 6 weeks until 8 weeks postintervention compared with that in the PBS-treated group. In addition, immunofluorescence analysis of CGRP, which is a marker of peptidergic nociceptive C nerve fibers (Li et al. 2020a, b), was performed to further compare the changes in nociceptive nerve fibers in subchondral bone from 4 weeks to 8 weeks in each group. As shown in Fig. 3D-E, after LSI surgery, there was a significant increase in CGRP at 4 weeks, which continued until 8 weeks, while few CGRP^+^ nociceptor fibres were detected in the subchondral bone in the control group (PBS injection without LSI surgery). The administration of ADSC-Exos alleviated CGRP + nerves in subchondral bone compared with those in the PBS group at 6 and 8 weeks after LSI model induction. Furthermore, Hypo-ADSC-Exos significantly ameliorated CGRP^+^ nociceptor fibres compared to those in the ADSC-Exo groups at 6 and 8 weeks, as indicated by immunofluorescence, which was consistent with the spinal pain behavioural tests. The Hypo-ADSC-Exo-treated group not only showed better pressure tolerance and less retraction frequency of the hind paw but also exhibited fewer CGRP^+^ nerves than the ADSC-Exo group, which indicated that hypoxia preconditioning could optimize the therapeutic effect of ADSC-Exos on the alleviation of symptomatic spinal pain caused by LFJ OA.

### In vivo tracing of Hypo-ADSC-Exos

As shown in Fig. 3F, PKH26-labelled exosomes could be taken up by chondrocytes in the cartilage and bone morrow cells in the subchondral bone of LFJ, which indicates that Hypo-ADSC-Exos could affect the cartilage and subchondral bone areas in LFJ after tail vein administration.

### Hypo-ADSC-Exos alleviate cartilage degeneration in the LFJ OA model

**Fig. 4:**
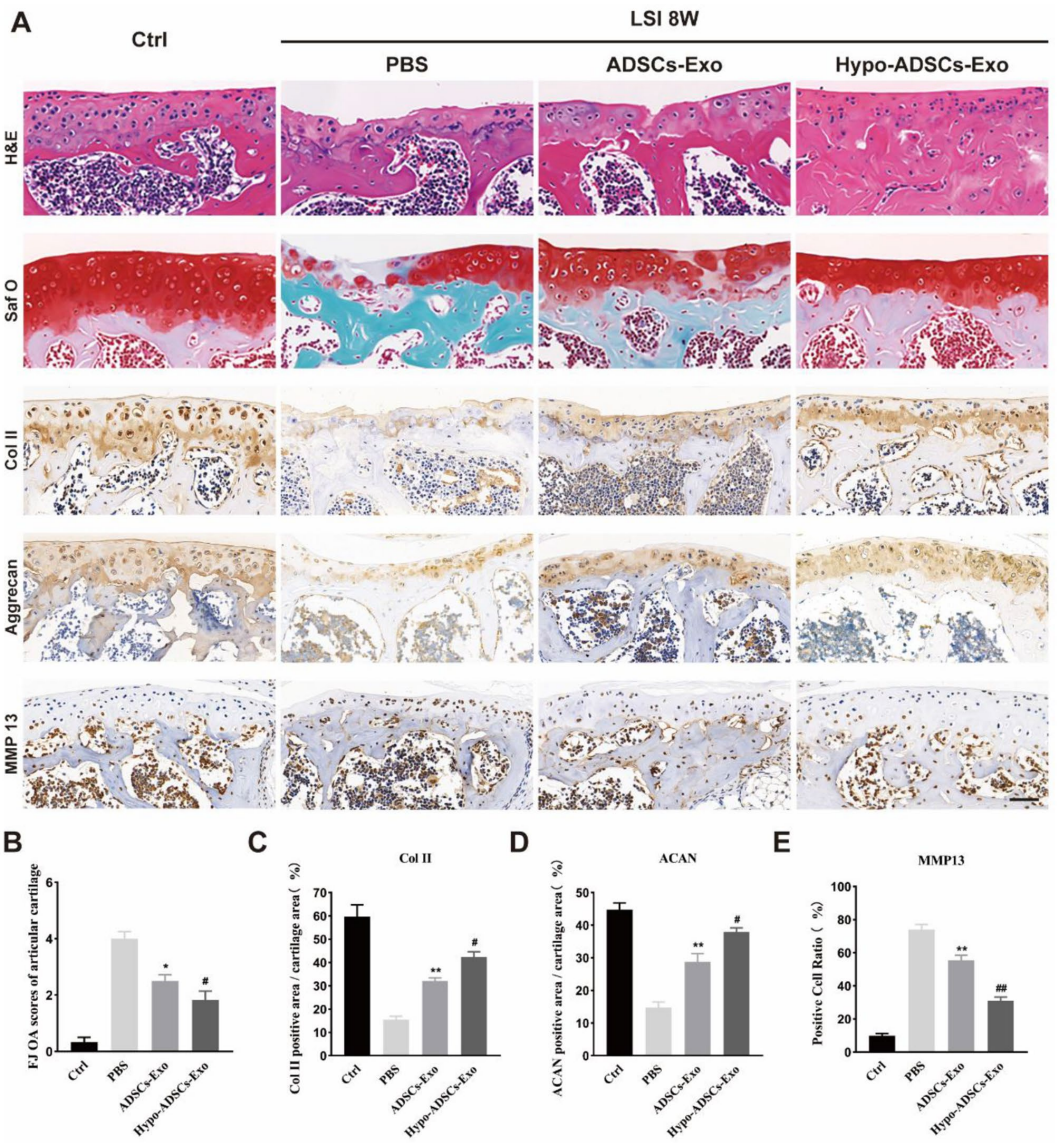
Hypo-ADSCs-Exo protect lumbar facet joint cartilage from degradation. (**A**) Representative histological images of LFJ cartilage with Hematoxylin-eosin (H&E) and Safranin O/Fast Green (top two) at 8 weeks post operation. Representative immunohistochemistry images of Collagen II, Aggrecan (middle two), and matrix metallopeptidase 13 (MMP13) (bottom) of LFJ cartilage. All images were captured under 40x objective lens. Scale bar=50 μm; (**B**) Semi-quantitative analysis of FJ OA scores of articular cartilages in (**A**); (**C-E**) Quantitative analysis of col II, Aggrecan, MMP-13 LFJ articular cartilage at 8 weeks post operation. All data are shown as the mean ±standard deviation (SD). n=6 per group. **p ＜ 0.01, compared with PBS treated group mice, ^#^p ＜ 0.05, ^##^p ＜ 0.01 compared with ADSCs-Exo mice. n=6 per group

We then examined the protective effects of Hypo-ADSC-Exos on cartilage in vivo. Gross morphological images of the superior articular process of the 5th lumbar vertebra were collected after H&E and Safranin O/Fast Green staining. As shown in Fig. 4A, the articular cartilage was clear with a smooth and intact surface in the control group. However, in the PBS-treated group, LSI surgery led to cartilage destruction, which was characterized by thinner and lightly stained cartilage, suggesting that LSI induced LFJ OA-like pathologies, including significant degeneration of facet joint cartilage. The administration of ADSC-Exos significantly improved the condition of articular cartilage and resulted in less proteoglycan loss than PBS treatment. Furthermore, histopathological analysis of LFJ cartilage at 8 weeks postsurgery demonstrated a marked reduction in the degree of LFJ cartilage degeneration, as determined by a semi-quantitative FJ scoring system (Wang et al. 2022), and there was less proteoglycan loss in the Hypo-ADSC-Exo group than the ADSC-Exo group (Fig. 4B). In addition to cartilage degeneration, synovial inflammation is a crucial indicator of the progression of lumbar facet joint osteoarthritis. As shown in Figure S1, the LSI model resulted in significant synovial hyperplasia and inflammatory cell infiltration in the synovium of the L4 facet joint. After ADSC-Exo administration, there was robust improvement in synovial inflammation compared with the PBS-treated group. Furthermore, exosomes obtained from hypoxic ADSCs exhibited better effects on synovial inflammation than ADSC-Exos.

In addition, immunohistochemical staining of LFJ cartilage showed increased expression of MMP13 and decreased expression of collagen II and aggrecan in the PBS group compared with the control group. ADSC-Exos normalized the expression of MMP-13, collagen II and aggrecan in LFJ cartilage in the ADSC-Exo-treated group relative to the PBS-treated group. Exosomes obtained from hypoxic ADSCs showed better results than ADSC-Exos (Fig. 4C-E), which indicated that the administration of Hypo-ADSC-Exos to LSI mice is a good choice.

### Hypo-ADSC-Exos restore the levels and distribution of collagen and proteoglycans (PGs) in degenerative lumbar facet joint cartilage

**Fig. 5:**
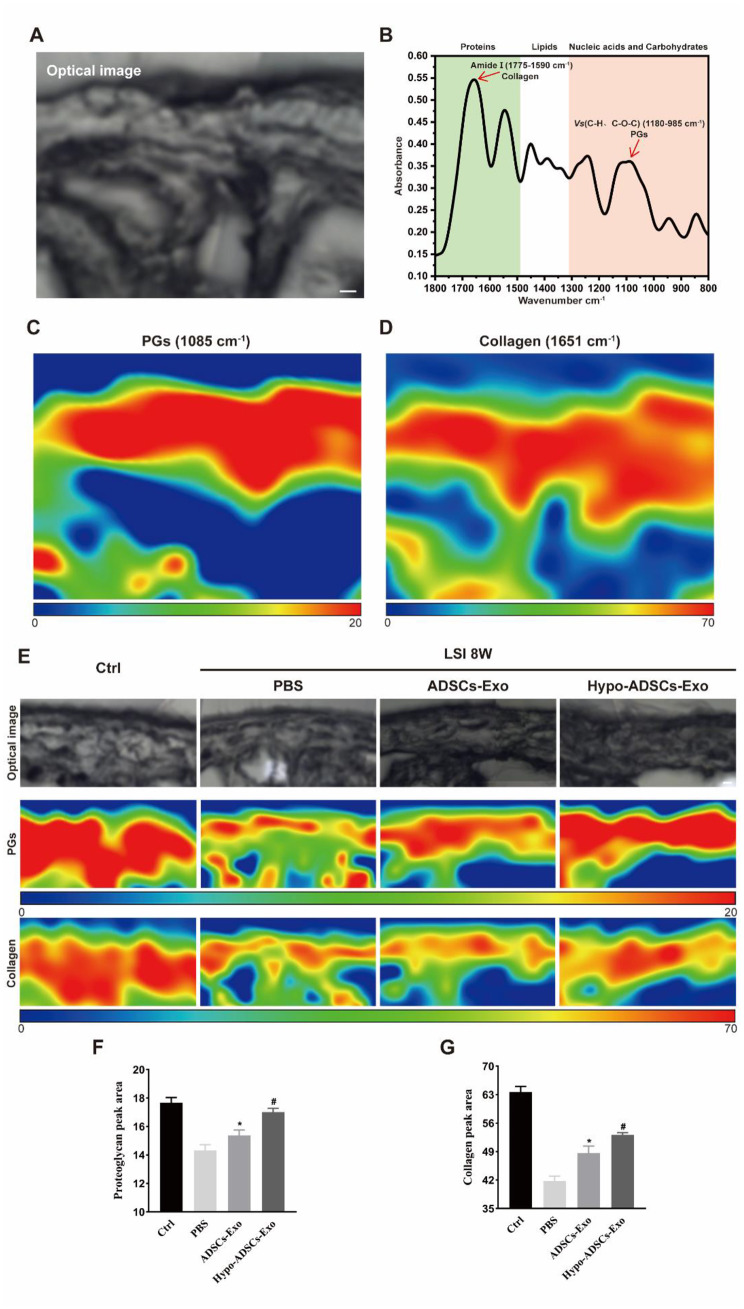
Hypo-ADSCs-Exo restores the contents of proteoglycans (PGs) and collagen in degenerative lumbar facet joint cartilage. (**A**) Synchrotron infrared imaging of lumbar facet joint showing the optical stereogram of the normal cartilage area; Scale bar, 20 μm (**B**) Representative infrared spectra. The absorption of the most important biomolecules is indicated. Red arrows point out the peaks of carbohydrate (1180-985 cm^-1^) and amide I (1775-1590 cm^-1^) which correlate with PGs and collagen respectively; (**C**) and (**D**) False-colored chemical mappings to record the distribution of PGs and collagen across cartilage area of lumbar facet joint according to their characteristic feature around 1805 cm^-1^ and 1651 cm^-1^ respectively. All chemical mappings were normalized to the same color scale for comparison purpose with red color representing the highest ratio and blue color the lowest ratio as shown below the figures; (**E**) Representative optical stereogram and chemical mappings of the collagen and PGs across cartilage area of lumbar facet joint among four groups; Optical images were captured under 32x objective lens. Scale bar, 20 μm (**F-G**) Quantitative analysis of PGs and collagen in (**E**). All data are shown as the mean ±standard deviation (SD). n=6 per group. *p ＜ 0.05, compared with PBS treated group mice, ^#^p ＜ 0.05, compared with ADSCs-Exo mice

To characterize the ECM in the cartilage layer of LFJ more precisely, we obtained optical images by light microscopy and spectra and chemical images by SR-FTIR (Fig. 5A and B). Peaks of carbohydrates (1180 − 985 cm^− 1^) and amide I (1775 − 1590 cm^− 1^), which correlate with collagen and PGs, respectively, were observed in normal cartilage. False-coloured chemical maps of SR-FTIR images show the levels and distribution of PGs and collagen. All chemical maps were normalized to the same colour scale for comparison purposes: red represents the highest ratio and blue represents the lowest ratio (Fig. 5C and D).

Then, optical images and spectroscopic maps of collagen and PGs (Fig. 5E) were obtained in the four groups by SR-FTIR. Compared to those in the PBS group, collagen and PG levels and distribution in LFJ cartilage in the ADSC-Exo group were restored (Fig. 5F and G). Furthermore, exosomes obtained from normoxic ADSCs showed less effects on collagen and PGs than hypoxic ADSC-Exos (Fig. 5F and G), which indicated that Hypo-ADSC-Exo treatment of FJ OA in LSI mice is a better method.

### Hypo-ADSC-Exos sustain coupled LFJ subchondral bone remodelling

**Fig. 6:**
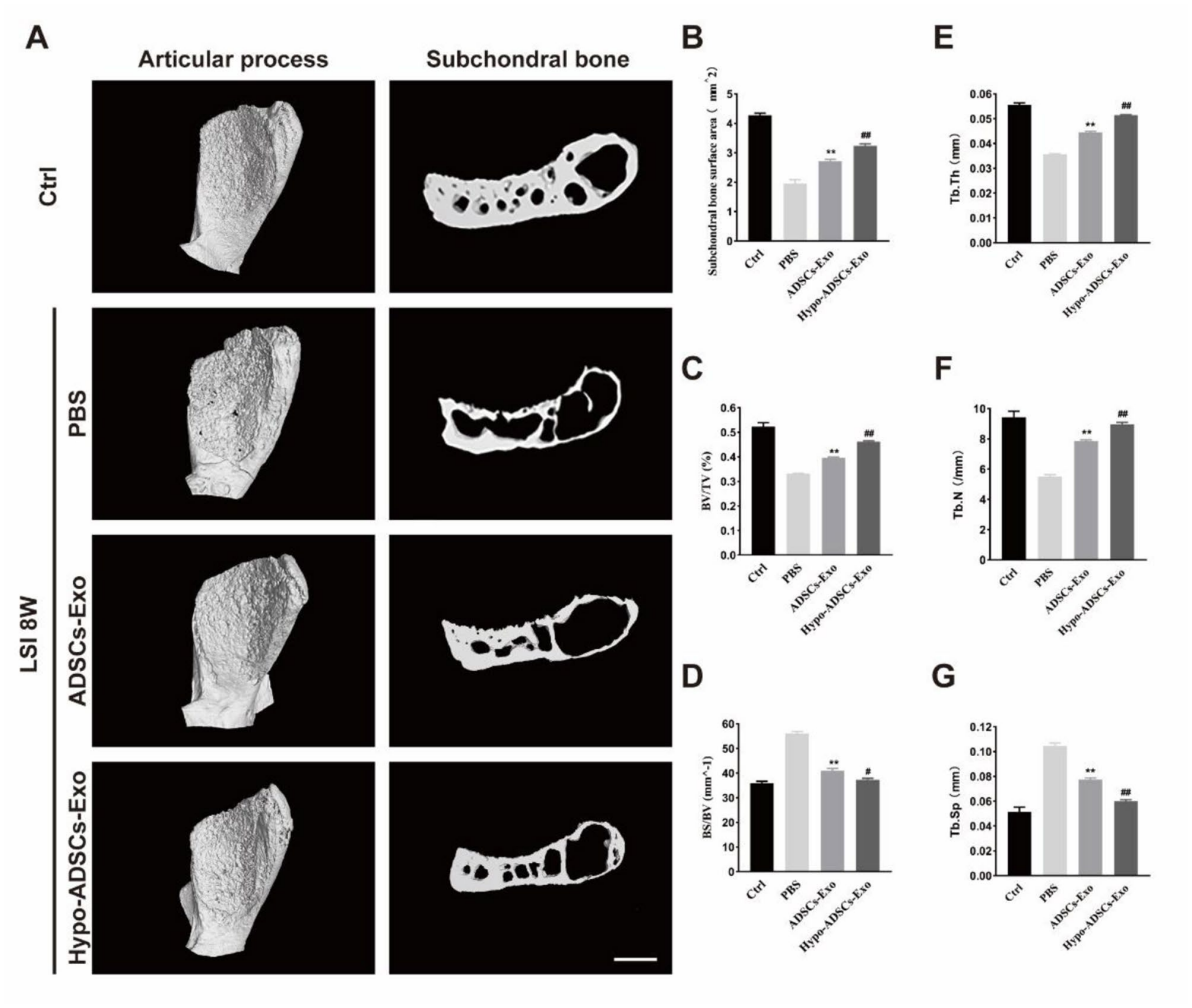
Hypo-ADSCs-Exo preserved the subchondral bone microarchitecture in LFJ OA. (**A**) 3D reconstructed X-ray microscope images of sub-chondral bone surface of the superior articular process and (left panel) corresponding sagittal microstructure (right panel) among the control, PBS or ADSCs-Exo and Hypo-ADSCs-Exo treated groups. Scale bar=200 μm; (**B**) – (**G**) Histomorphometry analysis of 3D images of the LFJ subchondral bone among four different groups. All data are shown as the mean ±standard deviation (SD). n=6 per group. **p＜0.01, compared with PBS treated group mice, ^#^p ＜ 0.05, ^##^p ＜ 0.01, compared with ADSCs-Exo mice

**Fig. 7:**
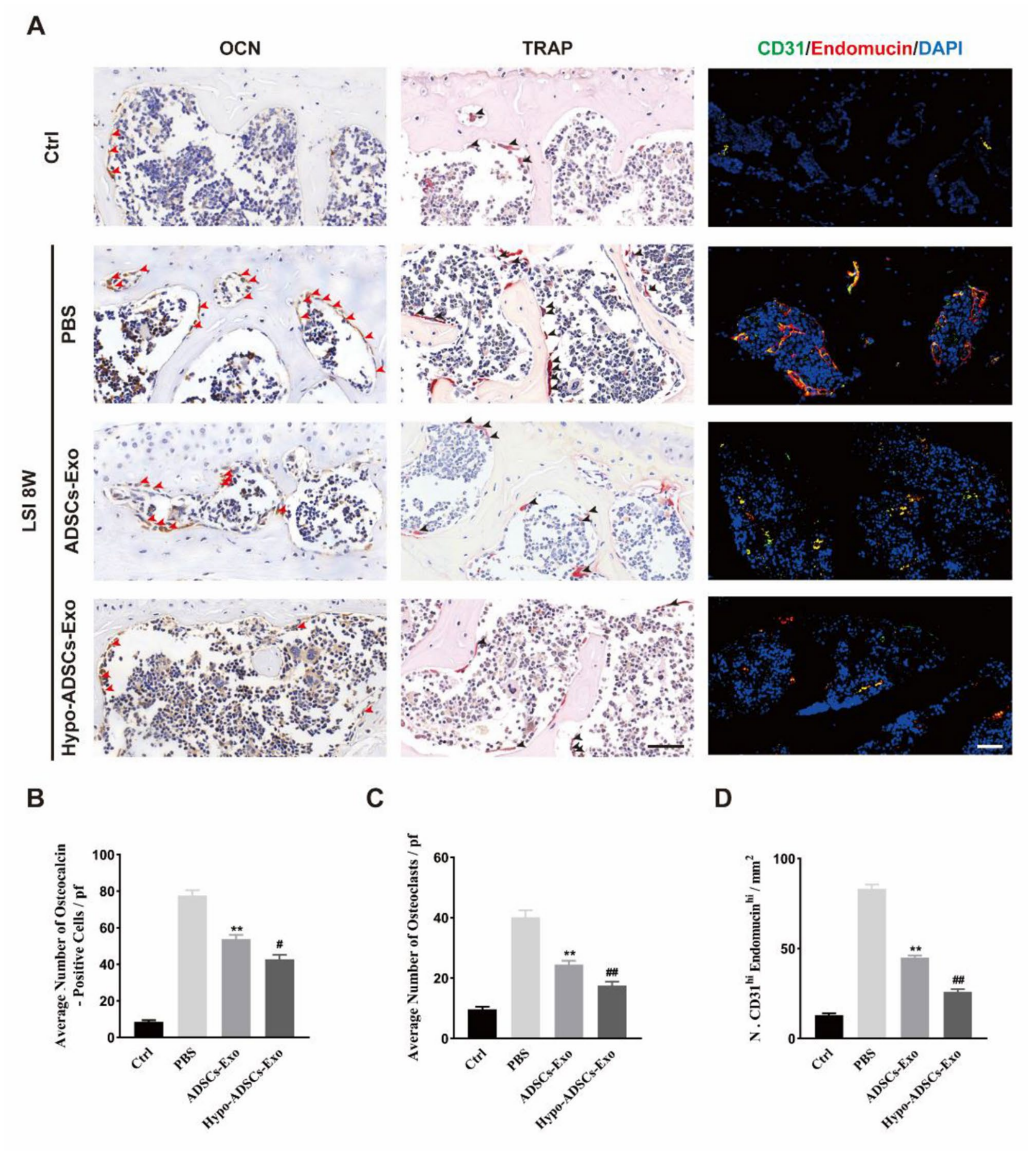
Hypo-ADSCs-Exo sustained coupled subchondral bone remodeling of LFJOA and attenuated the aberrant H-type vessels formation in subchondral bone. (**A**) Osteocalcin (OCN) staining (left panel) and TRAP staining (middle panel) in the subchondral bone of LFJ among different groups. Arrows point out the positive staining. Representative immunofluorescence of CD31 (Green), and Endomucin (Red) for H type vessel in the subchondral bone of LFJ among four groups (right panel). All images were captured under 40x objective lens. Scale bar=50 μm; (**B-C**) The statistical analysis of the ratio of OCN (**B**) and TRAP (**C**) positive cells in the subchondral bone of LFJ. (**D**) The statistical results of the double staining positive (CD31^+^ Endomucin^+^) cells in the subchondral bone of LFJ. All data are shown as the mean ±standard deviation (SD). n=6 per group. **p ＜ 0.01, compared with PBS treated group mice, ^#^p ＜ 0.05, ^##^p ＜ 0.01, compared with ADSCs-Exo mice

The effect of Hypo-ADSC-Exos on the structural changes in LFJ subchondral bone was analysed by a 3D X-ray microscope. As shown in Fig. 6A, the 3D images revealed significant loss of structural integrity in the LFJ subchondral bone in PBS-treated mice. Hypo-ADSC-Exo and ADSC-Exo treatment maintained the integrity of LFJ subchondral bone. According to the sagittal reconstruction image of the LFJ, the subchondral bone quantity in the PBS treatment group was decreased, and the trabecular structure of the subchondral bone was sparse, broken, and distorted. In contrast, Hypo-ADSC-Exo and ADSC-Exo administration significantly ameliorated subchondral bone loss and preserved trabecular bone quality (Fig. 6B-G). Specifically, ADSC-Exo treatment significantly reduced the BS/BV, decreased the trabecular space (Tb.Sp), and increased the subchondral bone surface area, BV/TV, and TB. Th, TB. N postsurgery relative to those in the PBS-treated group. In addition, Hypo-ADSC-Exos showed better coupled subchondral bone remodelling relative to ADSC-Exos (Fig. 6B-G). Consistently, the number of TRAP^+^ osteoclasts and OCN^+^ cells increased after LSI induction. The increase in cell populations was abrogated by the administration of ADSC-Exos. Notably, Hypo-ADSC-Exo treatment resulted in fewer OCN (+) and Trap (+) cells than ADSC-Exo treatment (Fig. 7A-C), which indicated that hypoxia enhanced the effects of ADSC-Exos on normalizing bone remodelling in LFJ subchondral bone by balancing bone resorption and bone formation.

### Hypo-ADSC-Exos abrogate aberrant H-type vessel formation in LFJ subchondral bone

H-type vessels are a specific subtype of vessel that couples angiogenesis with osteogenesis. In PBS-treated mice, aberrant H-type vessel formation was observed in the subchondral bone areas of LFJ and were characterized by an increase in CD31 and Endomucin double-positive cells (Fig. 7A and D). We examined the effect of Hypo-ADSC-Exos and ADSC-Exos on aberrant H-type vessel formation in LFJ subchondral bone and found a significant decrease in CD31 and Endomucin double -positive cells in subchondral bone relative to the PBS-treated groups. Compared with ADSC-Exo-treated mice, Hypo-ADSC-Exo administration significantly restored double-positive cells in the subchondral bone area of the LFJ. These results indicated that Hypo-ADSC-Exos abrogated aberrant H-type vessel formation in LFJ subchondral bone.

## Discussion

In this study, we explored the therapeutic effect of Hypo-ADSC-Exos and ADSC-Exos on LFJ OA. The results showed that hypoxia could enhance the effect of ADSC-Exos on optimizing the subchondral bone microarchitecture to prevent articular cartilage degeneration and alleviate symptomatic spinal pain-related behaviours in an LSI-induced LFJ OA model, providing a novel therapeutic solution for LFJ OA.

Exosome-based modification, which is an advanced strategy, has been gradually applied in the field of OA. Oxygen levels are crucial in controlling the proliferation and differentiation of MSCs (Xing et al. 2018). The evidence shows that hypoxia can promote the release of BMSC-Exos (Li et al. 2019). Gao et al. (2021) confirmed that Hypo-MSC-Exos may have a better effect by promoting endothelial cell proliferation and migration. Interestingly, other studies have reported that higher levels of molecules are loaded in secreted exosomes in response to hypoxia (Ren et al. 2019). However, no such study has been performed to delineate the effect of Hypo-ADSC-Exos on LFJ OA. Our results indicated that Hypo-ADSC-Exo-treated mice had higher quality subchondral bone, 3D morphological parameters and less articular cartilage degeneration than mice treated with ADSC-Exos. In addition, hypoxia enhanced the effect of ADSC-Exos on normalizing uncoupled subchondral bone remodelling by rebalancing bone resorption and bone formation in vivo. Aberrant H-type vessel formation was also effectively reduced by Hypo-ADSC-Exo treatment. These results indicated that Hypo-ADSC-Exos had a superior therapeutic effect compared with ADSC-Exos and offered a novel therapeutic strategy against LFJ OA.

Increasing evidence indicates that articular cartilage and subchondral bone are a functional unit during the onset of OA (Goldring and Goldring 2016). It has been reported that abnormal subchondral bone remodelling can precede articular cartilage degeneration (Zhang and Wen 2021). In addition, subchondral bone undergoes precise modelling and remodelling processes in response to changes in the mechanical loading environment (Burr and Gallant 2012). Balanced modelling and remodelling processes maintain the homeostasis of subchondral bone. Once mechanical loading changes, aberrant mechanical loading environment occurs in the LSI animal model, which affects the surface of the LFJ and can lead to the uncoupling of subchondral bone remodelling and an increase in osteoclasts in the LFJ, which is consistent with humans and mice with knee OA (Zheng et al. 2019). When exposed to aberrant mechanical loading due to abnormal subchondral bone remodelling, articular cartilage will undergo degradation and the loss of extracellular matrix. In our study, ADSC-Exos were administered by tail vein injection and internalized in the subchondral and articular cartilage areas, indicating that the therapeutic effect of exosomes not only involved cartilage metabolism but also subchondral bone coupling remodelling. We found that in ADSC-Exo-treated mice, the number of OCN- and Trap-positive cells reached a balance in subchondral bone area, indicating the re-establishment of coupled bone remodelling. In addition, ADSC-Exos inhibited articular cartilage degradation by promoting cartilage anabolism and preventing cartilage catabolism.

Recently, abnormal H-type vessel formation has been observed in subchondral bone and is a new pathological feature of OA (Peng et al. 2020). We observed the same phenotype in the LFJ OA model. The increase in the number of OCN- and TRAP-positive cells indicated that the bone turnover rate was accelerated in the subchondral bone in LFJ OA. Activated osteoclasts release multiple factors, such as PDGF-BB, and promote CD31^+^Endomucin^+^ type H vessel formation, which accelerates the bone turnover rate, erodes the cartilage matrix, and aggravates LFJ OA progression. H-type vessels were increased after LSI-induced LFJ OA but were similar to sham-operated controls when LSI mice were treated with ADSC-Exos. These results suggest that ADSC-Exos can attenuate OA progression by preventing pathological H-type vessel formation in the subchondral bone. In parallel with the pathological changes in subchondral bone, we also found that treatment with ADSC-Exos significantly optimized subchondral bone 3D morphological parameters, as shown by 3D X-ray microscopy scanning. Taken together, these data indicate that the normalization of subchondral bone remodelling in mice treated with ADSC-Exos further protects articular cartilage from degeneration by optimizing the microenvironment in the subchondral bone.

Apart from abnormal H-type vessels in subchondral bone associated with LFJ OA in the LSI model, aberrant sensory nerve innervation in subchondral bone has also been recognized as a source of osteoarthritic pain (Zhu et al. 2019). The microenvironment of subchondral bone in OA could be characterized by an elevated bone turnover rate, abnormal microstructures, aberrant H-type vessel formation, and sensory nerve innervation (Li et al. 2013). We hypothesize that the optimized microenvironment in subchondral bone treated with ADSC-Exos could inhibit aberrant sensory nerve innervation in subchondral bone. This may be the underlying mechanism by which ADSC-Exos exert beneficial effects on alleviating symptomatic spinal pain-related behaviours.

It is well documented that the development of plantar-related mechanical allodynia indicates secondary hypersensitivity in LBP animal models (Shuang et al. 2015; Kim et al. 2011, 2015; Millecamps et al. 2015). One study demonstrated that the murine sciatic nerve of mostly originated from the L3 and L4 DRG (dorsal root ganglia) by using retrograde tracing from the hind paw (Rigaud et al. 2008), highlighting the anatomical basis of mechanical hypersensitivity following LSI surgery. In the present study, our data showed that Hypo-ADSC-Exos significantly alleviated mechanical allodynia compared with ADSC-Exos in the LSI model, indicating that hypoxic preconditioning of ADSC-Exo ameliorated spinal pain. However, the exact molecular mechanism by which Hypo-ADSC-Exos can relieve spinal pain needs to be further examined.

The extracellular matrix (ECM) of LFJ cartilage is a complex of self-assembled macromolecules and is predominantly composed of collagen and proteoglycan (Xie et al. 2019). The current study was the first to examine the levels and distribution of collagen and proteoglycan in the cartilage of LFJ by using SR-FTIR, which makes it possible to link intrinsic structures with the levels in cartilage. This technique, which takes advantage of a synchrotron radiation source, is capable of examining the molecular chemistry of microstructures at ultra-spatial resolutions (Lumi et al. 2021). As a non-destructive method, SR-FTIR can provide information including the quantity, composition, structure, and distribution of chemical constituents of cartilage and could also be used to monitor changing levels after the administration of exosomes (Zhou et al. 2018).

Our present study has several limitations. First, the exact mechanism by which hypoxic ADSC-Exos improve LFJ degeneration and spinal pain is unclear. Future work should focus on exploring the molecular machinery. Second, we only examined the therapeutic effect of Hypo-ADSC-Exos for 8 weeks after LSI surgery, and a longer timeline should be investigated in future studies.

## Conclusion

Hypoxic preconditioning enhanced the therapeutic effects of ADSC-Exos on ameliorating pain and LFJ OA, suggesting that Hypo-ADSC-Exos are a promising therapeutic solution for LFJ OA treatment.

### Electronic supplementary material

Below is the link to the electronic supplementary material.

Supplementary Material 1

**Figure 1**. Identification of normoxia and hypoxia treated ADSCs-Exo. **(A)** The flow chart of isolation protocols for Hyo-ADSCs-Exo and ADSCs-Exo; **(B)** Morphology of ADSCs-Exo derived from hypoxic and normoxic conditions, as assessed by TEM; **(C)** The size distribution of Hypo-ADSCs-Exo and ADSCs-Exo; **(D)** Western blotting demonstrated the presence of exosomal surface markers CD81, CD63, and TSG101 between ADSCs-Exo and Hypo-ADSCs-Exo. **Figure 2** Schematic of lumbar spine instability (LSI) model establishment and diagram of timeline for examination postoperative among different treatment groups in vivo. **(A)** Schematic of lumbar spine instability (LSI) model establishment and diagram of timeline for examination postoperative among different treatment groups in vivo. **(A)** Schematic of LSI model establishment and red arrows indicate the facet joint osteoarthritis induced by LSI; **(B)** Group information and details; **(C)** Schedule time points for spinal pain tests, histological evaluation, immunohistochemi-stry, SR-FTIR, 3D X-ray microscopy analysis and immunofluorescence. **Figure 3**. Immunofluorescence of CGRP+ and exosomes uptake and quantitative analysis of spinal pain-related behavior tests among different treatment groups. (A-B) The hind-paw withdrawal frequency (PWF) responding to the Von-Frey filaments with 0.7 mN and 3,9 mN; **(C)** Pressure hyperalgesia of the lumbar spine. **(D)** Representative images of immunofluorescence of CGRP+ (A marker of nociceptor nerves, Red-Alexa Fluor® 594) in subchondral bone of LFJ in vivo under 40x objective lens in 4, 6 and 8 weeks. Scale bar, 100 µm. **(E)** Quantitative analysis of the percentage of CGRP + area in subchondral bone of LFJ. **(F)** Representative image of immunofluorescence of PKH26 labeled Hypo-ADSCs-Exo (PKH26^+^, Red) through tail vein administration in facet joint cartilage and subchondral bone of LFJ in vivo. The yellow arrows indicate that Hypo-ADSCs-Exo by administration of tail vein were internalized in cartilage zone and subchondral bone area. Scale bar, 100 µm. All images were captured under 40x objective lens. All data are shown as the mean ± standard deviation (SD). ^*^p<0.05, ^**^p<0.01, compared with PBS treated group mice, ^#^*p*<0.05, ^##^*p*<0.01, compared with ADSCs-Exo mice. n.s., non-significant. n = 6 per group. **Figure 4**. Hypo-ADSCs-Exo protect lumbar facet joint cartilage from degradation. **(A)** Representative histological images of LFJ cartilage with Hematoxylin-eosin (H&E) and Safranin O/Fast Green (top two) at 8 weeks post operation. Representative immunohistochemistry images of Collagen II, Aggrecan (middle two), and matrix metallopeptidase 13 (MMP13) (bottom) of LFJ cartilage. All images were captured under 40x objective lens. Scale bar = 50 µm; **(B)** Semi-quantitative analysis of FJ OA scores of articular cartilages in **(A)**; **(C-E)** Quantitative analysis of col II, Aggrecan, MMP-13 LFJ articular cartilage at 8 weeks post operation. All data are shown as the mean ± standard deviation (SD). n = 6 per group. ^**^p<0.01, compared with PBS treated group mice, ^#^*p*<0.05, ^##^*p*<0.01 compared with ADSCs-Exo mice. n = 6 per group. **Figure 5**. Hypo-ADSCs-Exo restores the contents of proteoglycans (PGs) and collagen in degenerative lumbar facet joint cartilage. **(A)** Synchrotron infrared imaging of lumbar facet joint showing the optical stereogram of the normal cartilage area; Scale bar, 20 µm **(B)** Representative infrared spectra. The absorption of the most important biomolecules is indicated. Red arrows point out the peaks of carbohydrate (1180 − 985 cm^− 1^) and amide I (1775 − 1590 cm^− 1^) which correlate with PGs and collagen respectively; **(C)** and **(D)** False-colored chemical mappings to record the distribution of PGs and collagen across cartilage area of lumbar facet joint according to their characteristic feature around 1805 cm^− 1^ and 1651 cm^− 1^ respectively. All chemical mappings were normalized to the same color scale for comparison purpose with red color representing the highest ratio and blue color the lowest ratio as shown below the figures; **(E)** Representative optical stereogram and chemical mappings of the collagen and PGs across cartilage area of lumbar facet joint among four groups; Optical images were captured under 32x objective lens. Scale bar, 20 µm **(F-G)** Quantitative analysis of PGs and collagen in **(E)**. All data are shown as the mean ± standard deviation (SD). n = 6 per group. ^*^p<0.05, compared with PBS treated group mice, ^#^*p*<0.05, compared with ADSCs-Exo mice. **Figure 6**. Hypo-ADSCs-Exo preserved the subchondral bone microarchitecture in LFJ OA. (A) 3D reconstructed X-ray microscope images of sub-chondral bone surface of the superior articular process and (left panel) corresponding sagittal microstructure (right panel) among the control, PBS or ADSCs-Exo and Hypo-ADSCs-Exo treated groups. Scale bar = 200 µm; **(B) – (G)** Histomorphometry analysis of 3D images of the LFJ subchondral bone among four different groups. All data are shown as the mean ± standard deviation (SD). n = 6 per group. ^**^p<0.01, compared with PBS treated group mice, ^#^*p*<0.05, ^##^*p*<0.01, compared with ADSCs-Exo mice. **Figure 7**. Hypo-ADSCs-Exo sustained coupled subchondral bone remodeling of LFJOA and attenuated the aberrant H-type vessels formation in subchondral bone. **(A**) Osteocalcin (OCN) staining (left panel) and TRAP staining (middle panel) in the subchondral bone of LFJ among different groups. Arrows point out the positive staining. Representative immunofluorescence of CD31 (Green), and Endomucin (Red) for H type vessel in the subchondral bone of LFJ among four groups (right panel). All images were captured under 40x objective lens. Scale bar = 50 µm; **(B-C)** The statistical analysis of the ratio of OCN **(B)** and TRAP **(C)** positive cells in the subchondral bone of LFJ. **(D)** The statistical results of the double staining positive (CD31^+^ Endomucin^+^) cells in the subchondral bone of LFJ. All data are shown as the mean ± standard deviation (SD). n = 6 per group. ^**^p<0.01, compared with PBS treated group mice, ^#^*p*<0.05, ^##^*p*<0.01, compared with ADSCs-Exo mice. **Figure S1** Hypo-ADSCs-Exo protect synovial inflammation of lumbar facet joint after LSI surgery. **(A)** Representative histological images of LFJ synovium with Hematoxylin-eosin (H&E) at 8 weeks post operation. All images were captured under 40x objective lens. Scale bar = 50 µm; **(B)** Quantitative analysis of synovial inflammation at 8 weeks post operation. All data are shown as the mean ± standard deviation (SD). n = 6 per group. ^*^p<0.05, compared with PBS treated group mice, ^#^*p*<0.05, compared with ADSCs-Exo mice. n = 6 per group.

Supplementary Material 3

## Data Availability

The data that support the findings of this study are available from the corresponding author upon reasonable request.

## Abbreviations

LFJ-OA: Lumbar Facet Joint Osteoarthritis
Hypo-ADSCs-Exo: Hypoxia treated adipose
mesenchymal: Stem cells
SR-FTIR: Synchrotron radiation-Fourier transform infrared spectroscopy
LSI: Lumbar Spinal Instability
LBP: Low Back Pain
OCT: Optimal cutting temperature
CGRP: Calcitonin Gene-Related Peptide positive
DRG: Dorsal root ganglia
