# Supplementary material for: Hypoxia-treated adipose mesenchymal stem cell-derived exosomes attenuate lumbar facet joint osteoarthritis
Source: Mol Med. 2023 Sep 5;29:120. doi: 10.1186/s10020-023-00709-3 (PMC10478461; doi:10.1186/s10020-023-00709-3)
Supplement: Supplementary file 1 — Supplementary Material 1 [file 10020_2023_709_MOESM1_ESM.docx]

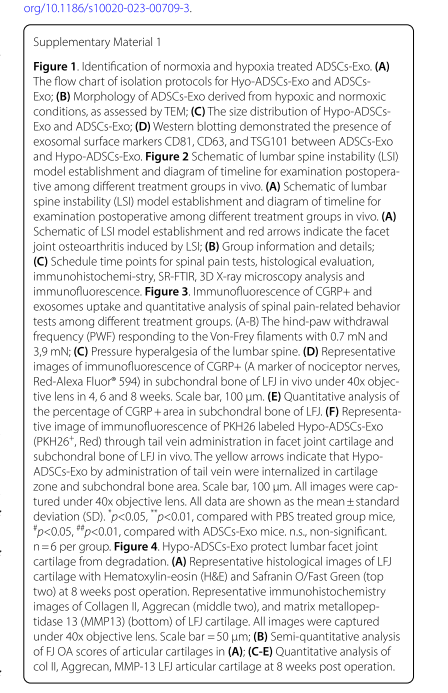


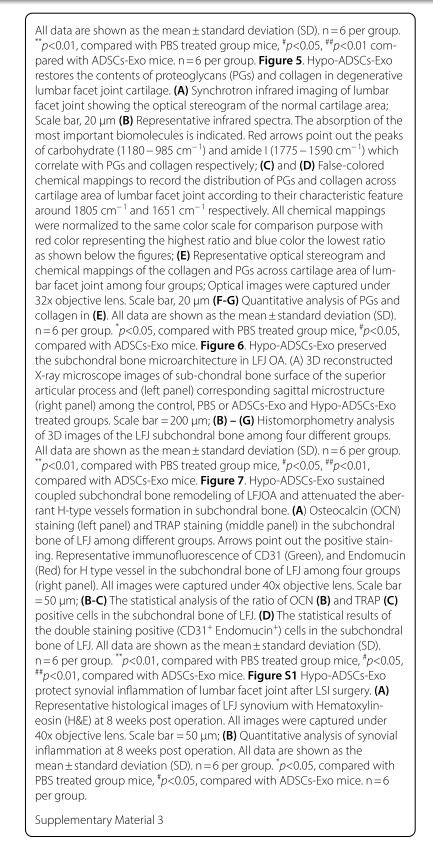


This part is not consistent with our figure legend part in Figure S1. Please correct it according to the figure legend below. Thank you.


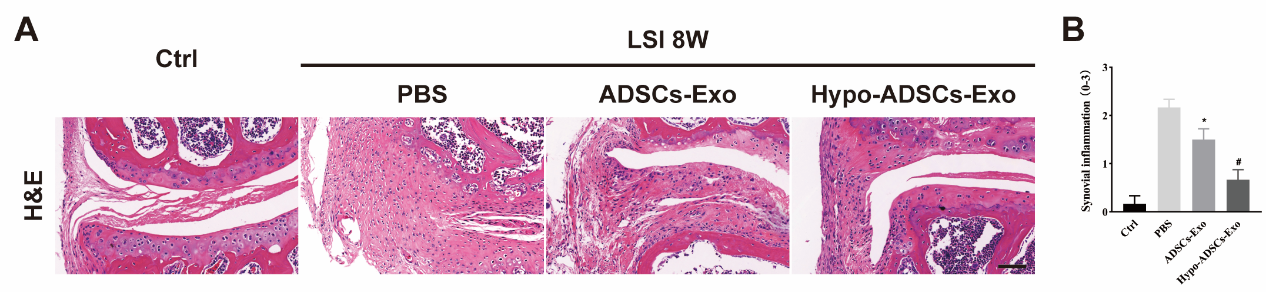


**Figure S1 Hypo-ADSCs-Exo protect synovial inflammation of lumbar facet joint after LSI surgery.** **(A)** Representative histological images of LFJ synovium with Hematoxylin-eosin (H&E) at 8 weeks post operation. All images were captured under 40x objective lens. Scale bar=50 μm; **(B)** Quantitative analysis of synovial inflammation at 8 weeks post operation. All data are shown as the mean ± standard deviation (SD). n=6 per group. ^*^*p*＜0.05, compared with PBS treated group mice, ^#^*p*＜0.05, compared with ADSCs-Exo mice. n=6 per group.
