# Supplementary material for: Hypoxia-treated adipose mesenchymal stem cell-derived exosomes attenuate lumbar facet joint osteoarthritis
Source: Mol Med. 2023 Sep 5;29:120. doi: 10.1186/s10020-023-00709-3 (PMC10478461; doi:10.1186/s10020-023-00709-3)
Supplement: Supplementary file 2 — Figure 1. Identification of normoxia and hypoxia treated ADSCs-Exo. (A) The flow chart of isolation protocols for Hyo-ADSCs-Exo and ADSCs-Exo; (B) Morphology of ADSCs-Exo derived from hypoxic and normoxic conditions, as assessed by TEM; (C) The size distribution of Hypo-ADSCs-Exo and ADSCs-Exo; (D) Western blotting demonstrated the presence of exosomal surface markers CD81, CD63, and TSG101 between ADSCs-Exo and Hypo-ADSCs-Exo. Figure 2 Schematic of lumbar spine instability (LSI) model establishment and diagram of timeline for examination postoperative among different treatment groups in vivo. (A) Schematic of lumbar spine instability (LSI) model establishment and diagram of timeline for examination postoperative among different treatment groups in vivo. (A) Schematic of LSI model establishment and red arrows indicate the facet joint osteoarthritis induced by LSI; (B) Group information and details; (C) Schedule time points for spinal pain tests, histological evaluation, immunohistochemi-stry, SR-FTIR, 3D X-ray microscopy analysis and immunofluorescence. Figure 3. Immunofluorescence of CGRP+ and exosomes uptake and quantitative analysis of spinal pain-related behavior tests among different treatment groups. (A-B) The hind-paw withdrawal frequency (PWF) responding to the Von-Frey filaments with 0.7 mN and 3,9 mN; (C) Pressure hyperalgesia of the lumbar spine. (D) Representative images of immunofluorescence of CGRP+ (A marker of nociceptor nerves, Red-Alexa Fluor® 594) in subchondral bone of LFJ in vivo under 40x objective lens in 4, 6 and 8 weeks. Scale bar, 100 µm. (E) Quantitative analysis of the percentage of CGRP + area in subchondral bone of LFJ. (F) Representative image of immunofluorescence of PKH26 labeled Hypo-ADSCs-Exo (PKH26+, Red) through tail vein administration in facet joint cartilage and subchondral bone of LFJ in vivo. The yellow arrows indicate that Hypo-ADSCs-Exo by administration of tail vein were internalized in cartilage zone and subchondral bone [file 10020_2023_709_MOESM2_ESM.docx]

**Figure 1**


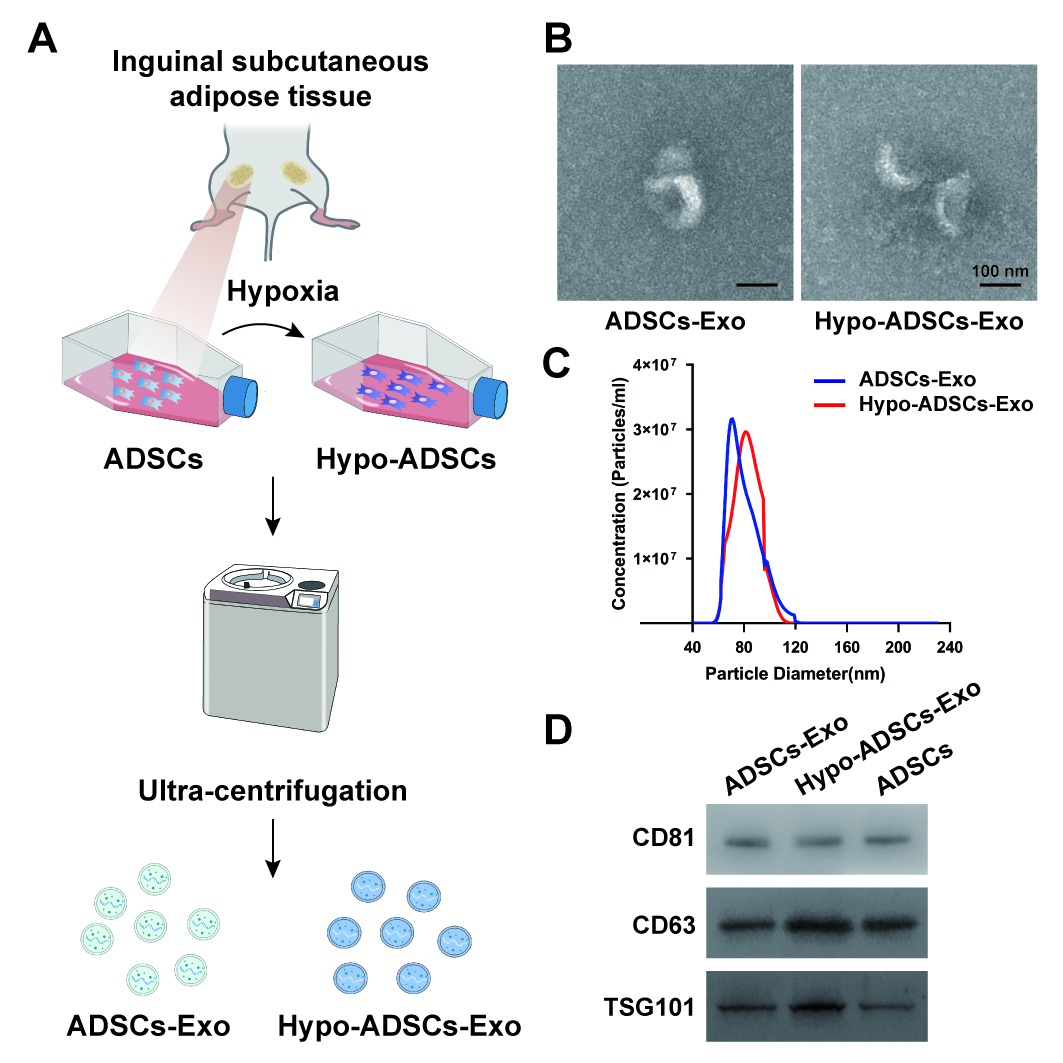


**Figure 1. Identification of normoxia and hypoxia treated ADSCs-Exo.** **(A)** The flow chart of isolation protocols for Hyo-ADSCs-Exo and ADSCs-Exo; **(B)** Morphology of ADSCs-Exo derived from hypoxic and normoxic conditions, as assessed by TEM; **(C)** The size distribution of Hypo-ADSCs-Exo and ADSCs-Exo; **(D)** Western blotting demonstrated the presence of exosomal surface markers CD81, CD63, and TSG101 between ADSCs-Exo and Hypo-ADSCs-Exo.

**Figure 2**


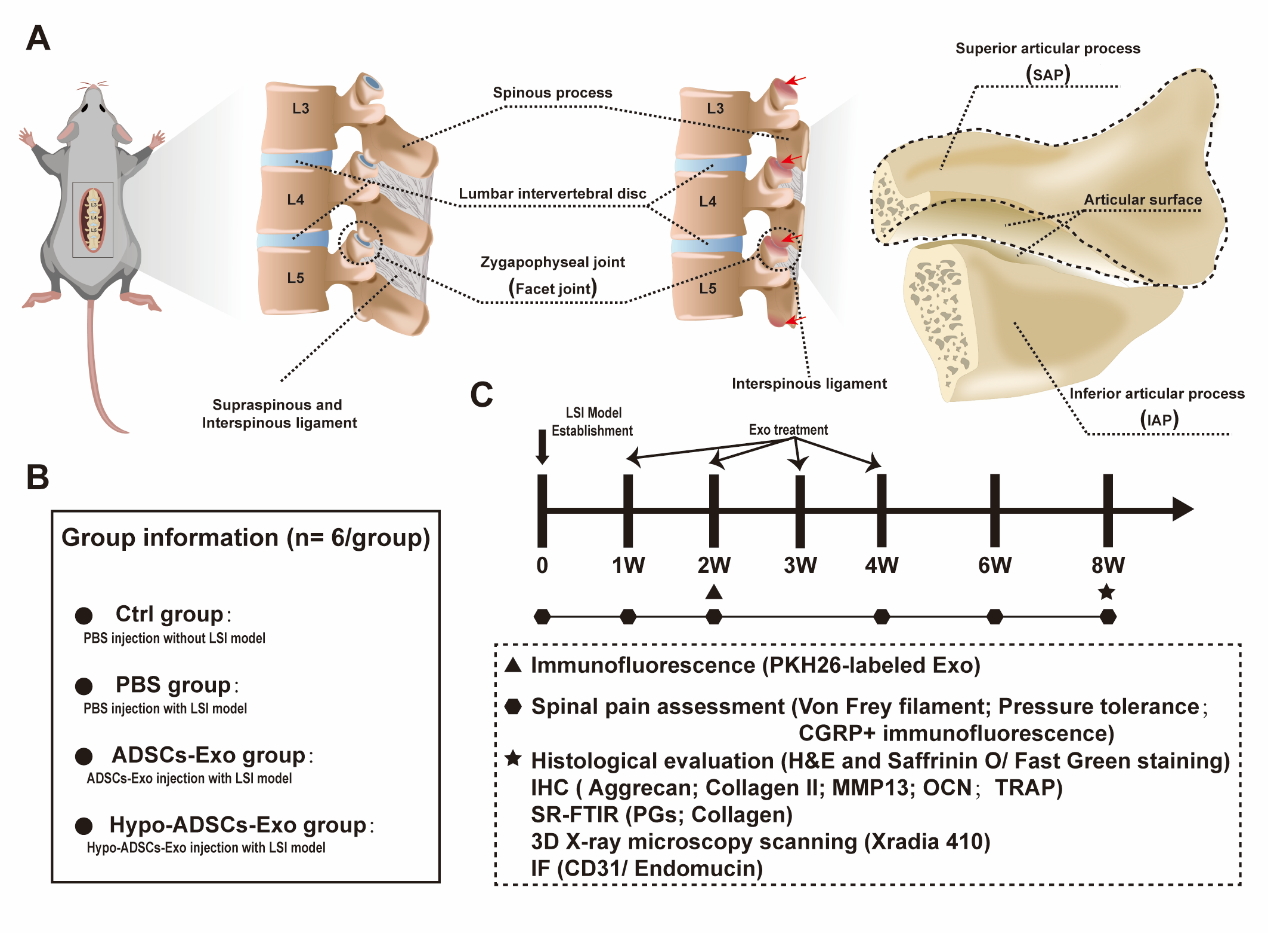


**Figure 2 Schematic of lumbar spine instability (LSI) model establishment and diagram of timeline for examination postoperative among different treatment groups in vivo. (A)** Schematic of LSI model establishment and red arrows indicate the facet joint osteoarthritis induced by LSI; **(B)** Group information and details; **(C)** Schedule time points for spinal pain tests, histological evaluation, immunohistochemi

-stry, SR-FTIR, 3D X-ray microscopy analysis and immunofluorescence.

**Figure 3**


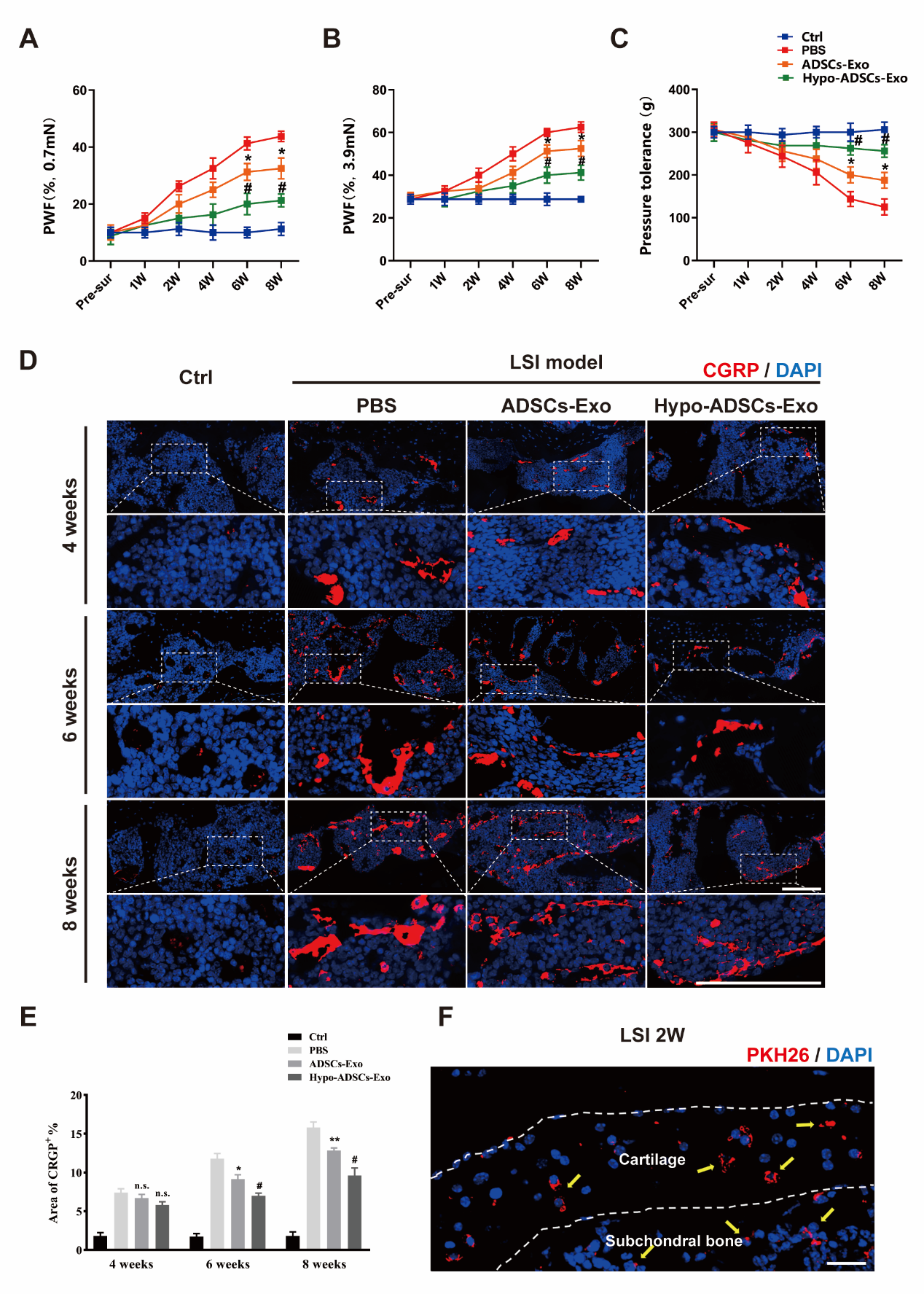


**Figure 3. Immunofluorescence of CGRP^+^ and exosomes uptake and quantitative analysis of spinal pain-related behavior tests among different treatment groups. (A-B)** The hind-paw withdrawal frequency (PWF) responding to the Von-Frey filaments with 0.7 mN and 3,9 mN; **(C)** Pressure hyperalgesia of the lumbar spine. **(D)** Representative images of immunofluorescence of CGRP+ (A marker of nociceptor nerves, Red-Alexa Fluor® 594) in subchondral bone of LFJ in vivo under 40x objective lens in 4, 6 and 8 weeks. Scale bar, 100 μm. **(E)** Quantitative analysis of the percentage of CGRP+ area in subchondral bone of LFJ. **(F)** Representative image of immunofluorescence of PKH26 labeled Hypo-ADSCs-Exo (PKH26^+^, Red) through tail vein administration in facet joint cartilage and subchondral bone of LFJ in vivo. The yellow arrows indicate that Hypo-ADSCs-Exo by administration of tail vein were internalized in cartilage zone and subchondral bone area. Scale bar, 100 μm. All images were captured under 40x objective lens. All data are shown as the mean ±standard deviation (SD). ^*^*p*＜0.05, ^**^*p*＜0.01, compared with PBS treated group mice, ^#^*p*＜0.05, ^##^*p*＜0.01, compared with ADSCs-Exo mice. n.s., non-significant. n=6 per group.

**Figure 4**


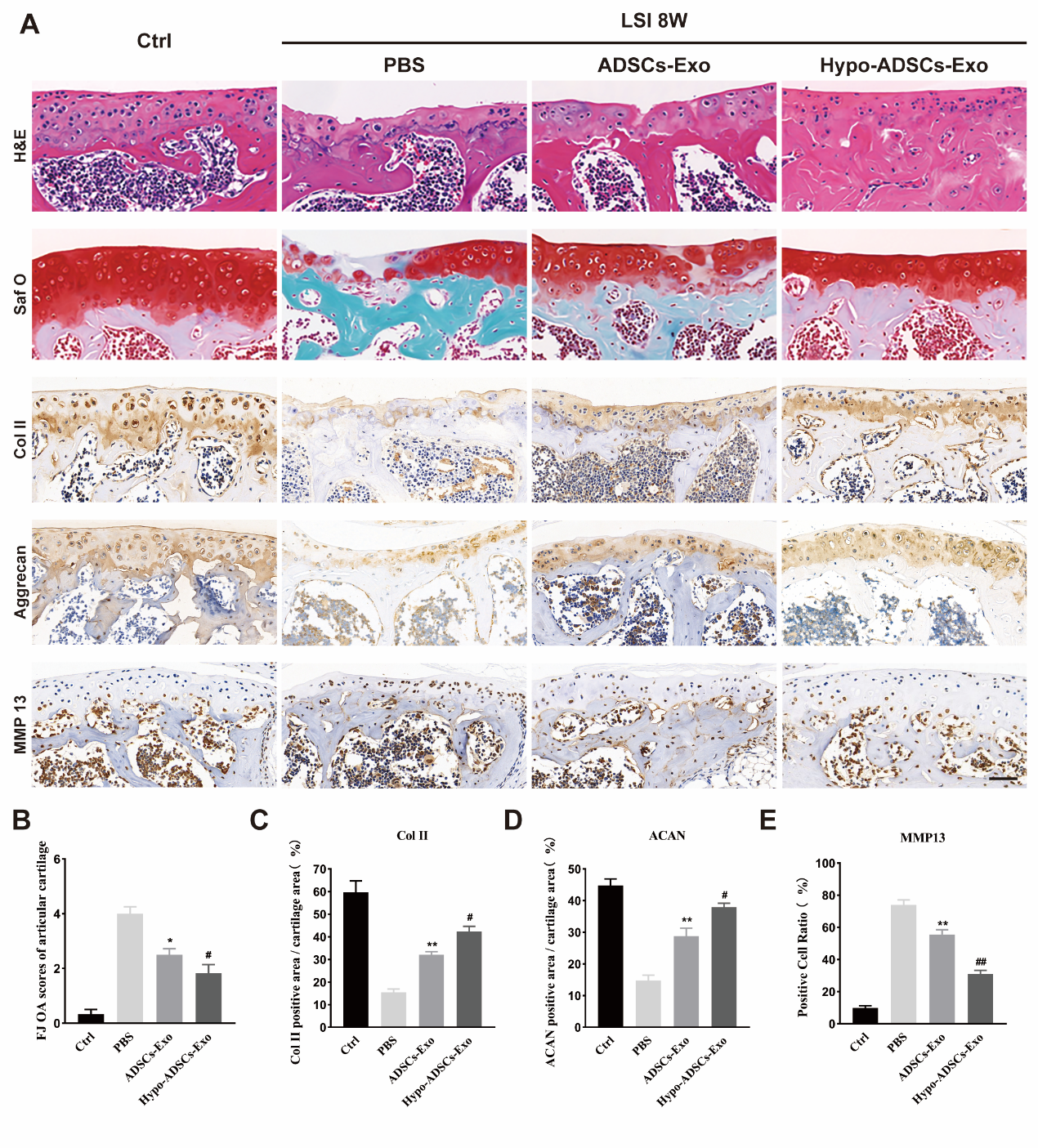


**Figure 4. Hypo-ADSCs-Exo protect lumbar facet joint cartilage from degradation.** **(A)** Representative histological images of LFJ cartilage with Hematoxylin-eosin (H&E) and Safranin O/Fast Green (top two) at 8 weeks post operation. Representative immunohistochemistry images of Collagen II, Aggrecan (middle two), and matrix metallopeptidase 13 (MMP13) (bottom) of LFJ cartilage. All images were captured under 40x objective lens. Scale bar=50 μm; **(B)** Semi-quantitative analysis of FJ OA scores of articular cartilages in **(A)**; **(C-E)** Quantitative analysis of col II, Aggrecan, MMP-13 LFJ articular cartilage at 8 weeks post operation. All data are shown as the mean ±standard deviation (SD). n=6 per group. ^**^*p*＜0.01, compared with PBS treated group mice, ^#^*p*＜0.05, ^##^*p*＜0.01 compared with ADSCs-Exo mice. n=6 per group.

**Figure 5**


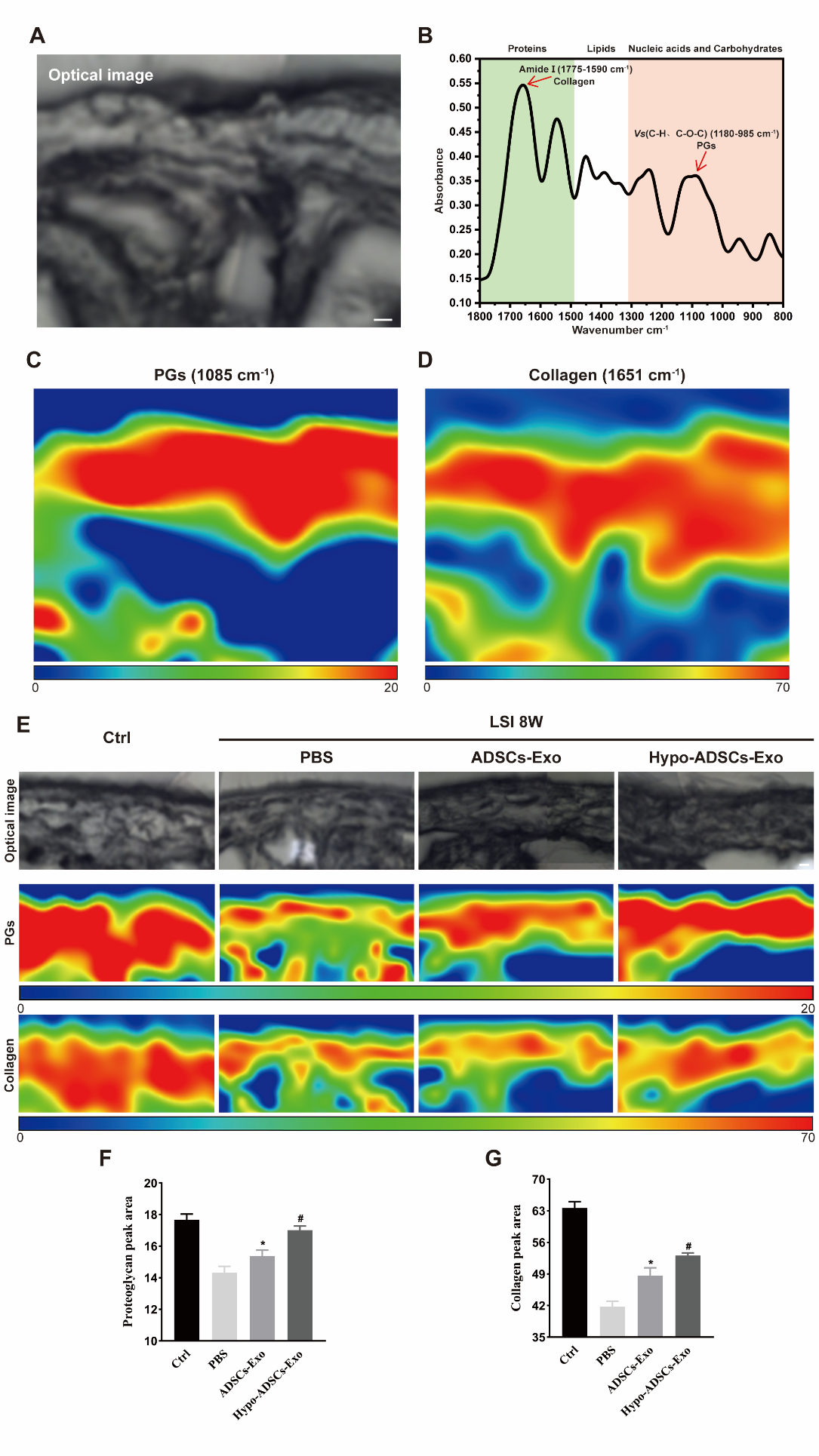


**Figure 5. Hypo-ADSCs-Exo restores the contents of proteoglycans (PGs) and collagen in degenerative lumbar facet joint cartilage. (A)** Synchrotron infrared imaging of lumbar facet joint showing the optical stereogram of the normal cartilage area; Scale bar, 20 μm **(B)** Representative infrared spectra. The absorption of the most important biomolecules is indicated. Red arrows point out the peaks of carbohydrate (1180-985 cm^-1^) and amide I (1775-1590 cm^-1^) which correlate with PGs and collagen respectively; **(C)** and **(D)** False-colored chemical mappings to record the distribution of PGs and collagen across cartilage area of lumbar facet joint according to their characteristic feature around 1805 cm^-1^ and 1651 cm^-1^ respectively. All chemical mappings were normalized to the same color scale for comparison purpose with red color representing the highest ratio and blue color the lowest ratio as shown below the ﬁgures; **(E)** Representative optical stereogram and chemical mappings of the collagen and PGs across cartilage area of lumbar facet joint among four groups; Optical images were captured under 32x objective lens. Scale bar, 20 μm **(F-G)** Quantitative analysis of PGs and collagen in **(E)**. All data are shown as the mean ±standard deviation (SD). n=6 per group. ^*^*p*＜0.05, compared with PBS treated group mice, ^#^*p*＜0.05, compared with ADSCs-Exo mice.

**Figure 6**


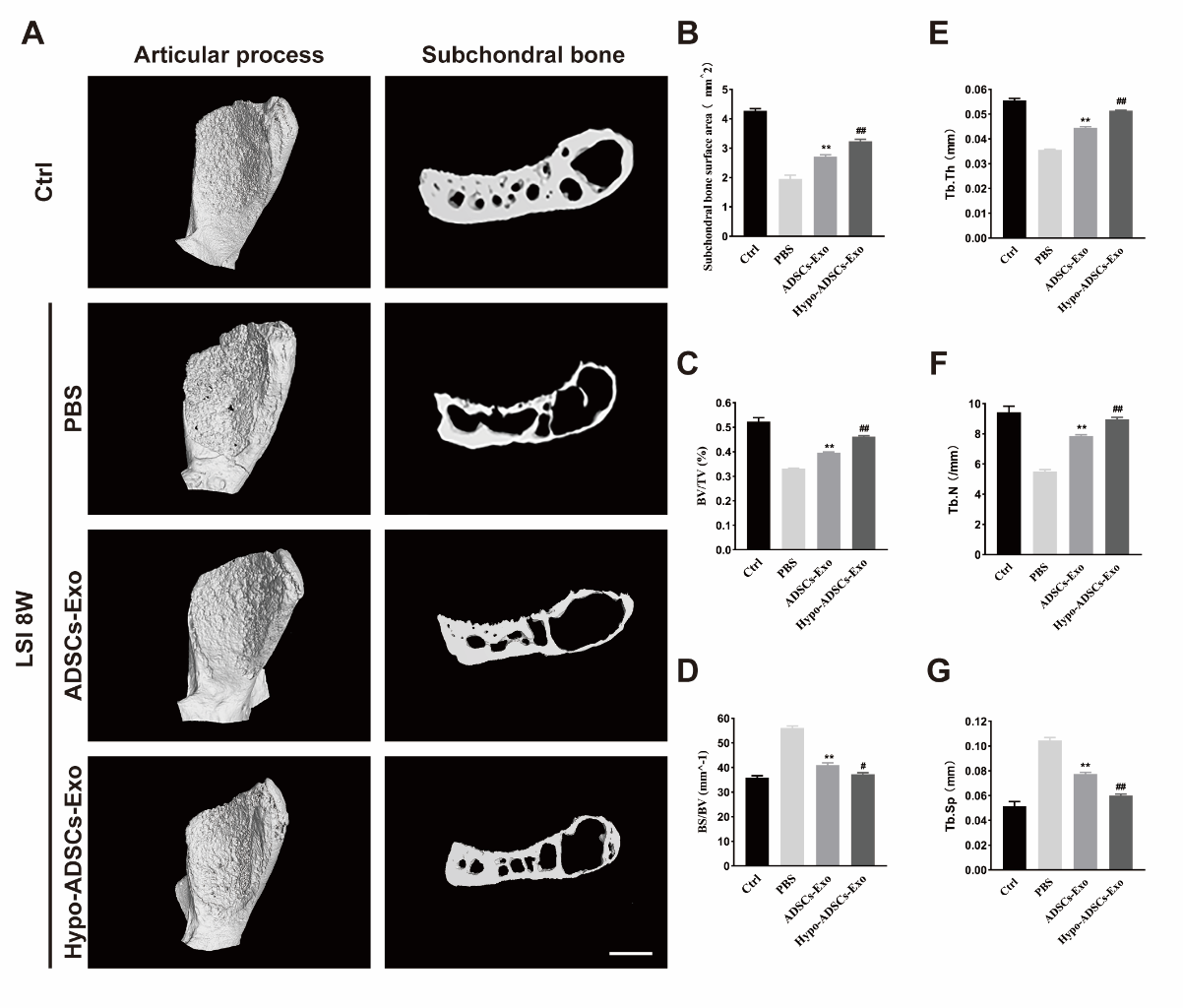


**Figure 6. Hypo-ADSCs-Exo preserved the subchondral bone microarchitecture in LFJ OA.** **(A)** 3D reconstructed X-ray microscope images of sub-chondral bone surface of the superior articular process and (left panel) corresponding sagittal microstructure (right panel) among the control, PBS or ADSCs-Exo and Hypo-ADSCs-Exo treated groups. Scale bar=200 μm; **(B) – (G)** Histomorphometry analysis of 3D images of the LFJ subchondral bone among four different groups. All data are shown as the mean ±standard deviation (SD). n=6 per group. ^**^*p*＜0.01, compared with PBS treated group mice, ^#^*p*＜0.05, ^##^*p*＜0.01, compared with ADSCs-Exo mice.

**Figure 7**


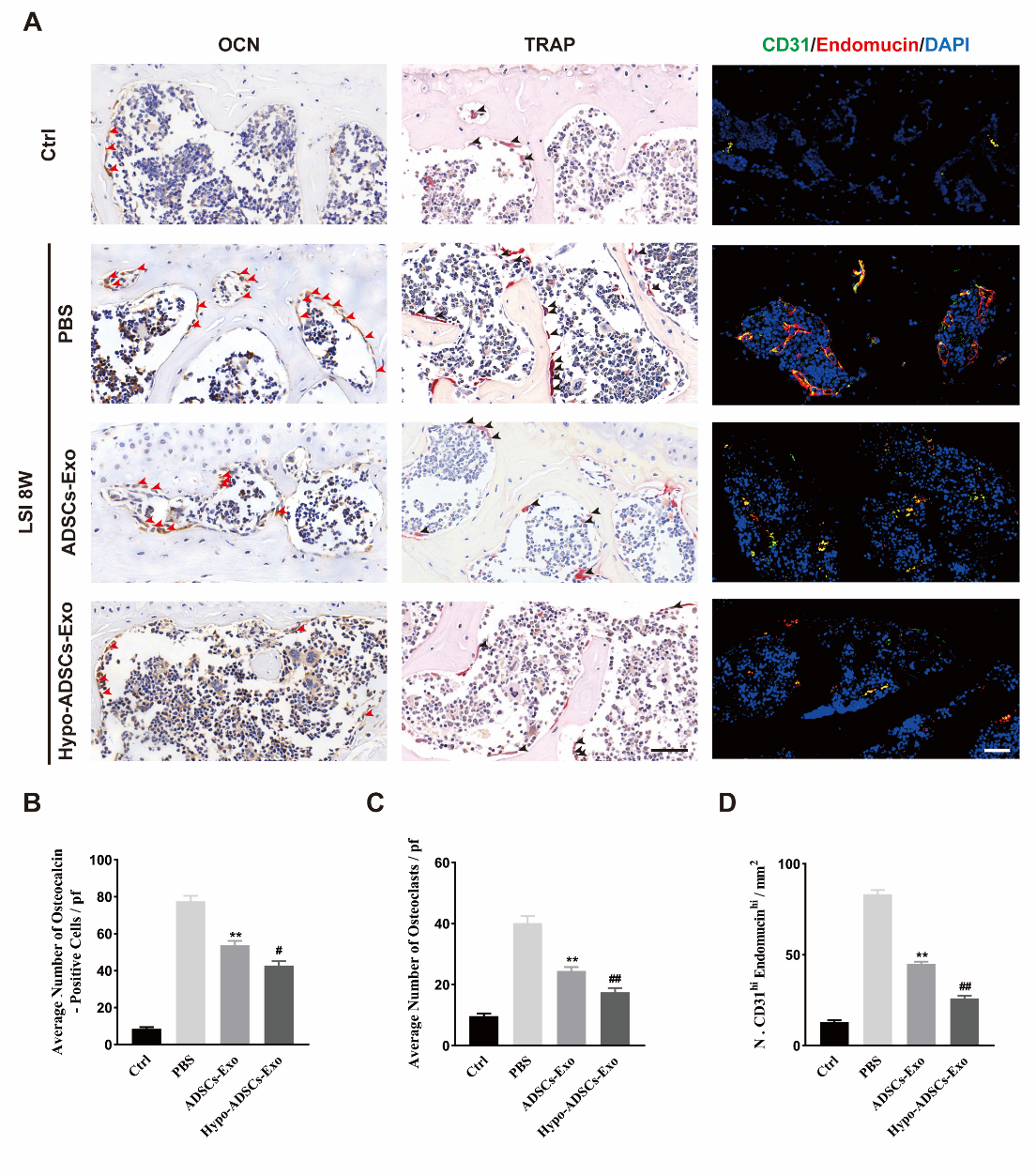


**Figure 7. Hypo-ADSCs-Exo sustained coupled subchondral bone remodeling of LFJOA and attenuated the aberrant H-type vessels formation in subchondral bone.** **(A)** Osteocalcin (OCN) staining (left panel) and TRAP staining (middle panel) in the subchondral bone of LFJ among different groups. Arrows point out the positive staining. Representative immunofluorescence of CD31 (Green), and Endomucin (Red) for H type vessel in the subchondral bone of LFJ among four groups (right panel). All images were captured under 40x objective lens. Scale bar=50 μm; **(B-C)** The statistical analysis of the ratio of OCN **(B)** and TRAP **(C)** positive cells in the subchondral bone of LFJ. **(D)** The statistical results of the double staining positive (CD31^+^ Endomucin^+^) cells in the subchondral bone of LFJ. All data are shown as the mean ±standard deviation (SD). n=6 per group. ^**^*p*＜0.01, compared with PBS treated group mice, ^#^*p*＜0.05, ^##^*p*＜0.01, compared with ADSCs-Exo mice.


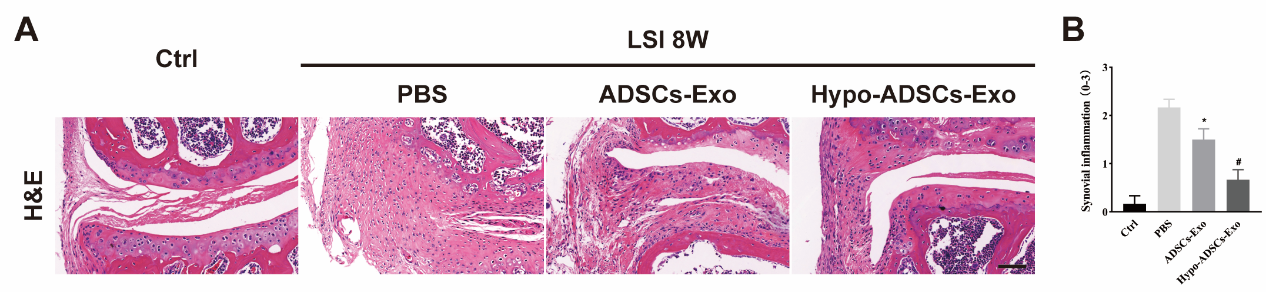


**Figure S1 Hypo-ADSCs-Exo protect synovial inflammation of lumbar facet joint after LSI surgery.** **(A)** Representative histological images of LFJ synovium with Hematoxylin-eosin (H&E) at 8 weeks post operation. All images were captured under 40x objective lens. Scale bar=50 μm; **(B)** Quantitative analysis of synovial inflammation at 8 weeks post operation. All data are shown as the mean ± standard deviation (SD). n=6 per group. ^*^*p*＜0.05, compared with PBS treated group mice, ^#^*p*＜0.05, compared with ADSCs-Exo mice. n=6 per group.
