# Supplementary material for: Hypoxia-treated adipose mesenchymal stem cell-derived exosomes attenuate lumbar facet joint osteoarthritis
Source: Mol Med. 2023 Sep 5;29:120. doi: 10.1186/s10020-023-00709-3 (PMC10478461; doi:10.1186/s10020-023-00709-3)
Supplement: Supplementary file 3 — Supplementary Material 3 [file 10020_2023_709_MOESM3_ESM.docx]

Dear editor,

We really appreciated for allowing us to third-revise our manuscript. We also thank the reviews for their thoughtful and constructive comments regarding to our manuscript. These comments are valuable and helpful for improving the quality of our paper.

Based on the comments the reviews provided, we uploaded the file of revised manuscript in which all the changes were highlighted in yellow background. We hope that the reviewers will find our edited manuscript satisfactory, and we are willing to finish the revised version of the manuscript including any further suggestion that the reviewers may have.

We look forward to hearing from you regarding our submission.

Thank you and best regards.

Chunyue Duan, PhD, MD

Professor,

Department of Spine Surgery,

Xiangya Hospital,

Central South University,

Changsha, Hunan, 410008,

China

Reviewer reports:

Reviewer #1: This manuscript has been improved, but an important flaw remains and must be addressed.

1. Line 197-199: In this study the authors utilized a semiquantitative FJOA scoring system published in JBJS, rather than an OARSI score. Please change "The OARSI score, which is a semiquantitative FJ OA scoring system, was used to evaluate articular cartilage as previously described (39)…" to "A semiquantitative FJOA scoring system was used to evaluate articular cartilage changes as previously described (39)…".

**Response：**Thank you very much for your great contribution to the improvement of our manuscript. We have changed ‘The OARSI score, which is a semiquantitative FJ OA scoring system, was used to evaluate articular cartilage as previously described (39)…’ to ‘A semiquantitative FJOA scoring system was used to evaluate articular cartilage changes as previously described (39)…’ according to your professional advices. Meanwhile, we have made modification of Figure 4B by using the semi-quantitative FJ OA scoring system rather than OARSI scores. Once again, we appreciate your efforts in improvement of our revised manuscript. (Line 197-198, Line 311-312, in yellow line)
